# Influence of host phylogeny and water physicochemistry on microbial assemblages of the fish skin microbiome

**DOI:** 10.1093/femsec/fiae021

**Published:** 2024-02-16

**Authors:** Ashley G Bell, Jamie McMurtrie, Luis M Bolaños, Jo Cable, Ben Temperton, Charles R Tyler

**Affiliations:** College of Life and Environmental Sciences, The University of Exeter, Exter, Devon EX4 4QD, United Kingdom; Sustainable Aquaculture Futures, The University of Exeter, Exter, Devon EX4 4QD, United Kingdom; College of Life and Environmental Sciences, The University of Exeter, Exter, Devon EX4 4QD, United Kingdom; Sustainable Aquaculture Futures, The University of Exeter, Exter, Devon EX4 4QD, United Kingdom; College of Life and Environmental Sciences, The University of Exeter, Exter, Devon EX4 4QD, United Kingdom; School of Biosciences, Cardiff University, Cardiff CF10 3AX, United Kingdom; College of Life and Environmental Sciences, The University of Exeter, Exter, Devon EX4 4QD, United Kingdom; College of Life and Environmental Sciences, The University of Exeter, Exter, Devon EX4 4QD, United Kingdom; Sustainable Aquaculture Futures, The University of Exeter, Exter, Devon EX4 4QD, United Kingdom

**Keywords:** 16S, aquaculture, meta-analysis, microbiota, phylosymbiosis, physicochemical factors, V4

## Abstract

The skin of fish contains a diverse microbiota that has symbiotic functions with the host, facilitating pathogen exclusion, immune system priming, and nutrient degradation. The composition of fish skin microbiomes varies across species and in response to a variety of stressors, however, there has been no systematic analysis across these studies to evaluate how these factors shape fish skin microbiomes. Here, we examined 1922 fish skin microbiomes from 36 studies that included 98 species and nine rearing conditions to investigate associations between fish skin microbiome, fish species, and water physiochemical factors. Proteobacteria, particularly the class Gammaproteobacteria, were present in all marine and freshwater fish skin microbiomes. *Acinetobacter, Aeromonas, Ralstonia, Sphingomonas* and *Flavobacterium* were the most abundant genera within freshwater fish skin microbiomes, and *Alteromonas, Photobacterium, Pseudoalteromonas, Psychrobacter* and *Vibrio* were the most abundant in saltwater fish. Our results show that different culturing (rearing) environments have a small but significant effect on the skin bacterial community compositions. Water temperature, pH, dissolved oxygen concentration, and salinity significantly correlated with differences in beta-diversity but not necessarily alpha-diversity. To improve study comparability on fish skin microbiomes, we provide recommendations for approaches to the analyses of sequencing data and improve study reproducibility.

## Teaser

Meta-analysis reveals interrelationships between fish species, water physicochemistry, rearing conditions and microbiota in the skin of salt and freshwater fish. Created with BioRender.com.

## Introduction

Bacteria are ubiquitous in nature and their abundance and community structure are influenced by a variety of biological, physical, and chemical factors (Thompson et al. 2017). There is a growing interest in characterizing the bacterial communities associated with higher eukaryotic hosts, termed microbiomes (Ursell et al. 2012, Byrd et al. 2018). Many of these bacterial communities form symbiotic relationships with their host, conferring benefits including facilitating pathogen exclusion, immune system priming, and nutrient degradation, all of which ultimately enhance host fitness (Belkaid and Hand 2014, Chiu et al. 2017, Pickard et al. 2017, Hou et al. 2022). Gnotobiotic animals grown in sterile laboratory conditions with limited microbiota demonstrate reduced resilience to disease and poorer health outcomes (Williams 2014, Tlaskalova-Hogenova et al. 2015). This highlights the importance of research on microbiomes and their relationship with their host organism to optimize animal health. To date, most research has focused on gut microbiomes in domesticated mammals (47.9%), principally because of the interest in their roles in human health and livestock production (Pascoe et al. 2017), yet mammals constitute less than 10% of total vertebrate diversity (IUCN Red List 2022).

Fish encompass 50% of all vertebrate diversity (IUCN Red List 2022), and their importance to global food security and ability to live in a wide range of different habitats highlights the need to better characterize the microbial communities in fish given that their importance for host health. There has been a recent and increasing interest in understanding how fish microbiomes affect growth and health in aquaculture Most of these studies have focused primarily on gut health and growth (Talwar et al. 2018, Huang et al. 2020, Legrand et al. 2020), including the effects of feeds (Karlsen et al. 2022), and associations with disease and immunological defence (Shi et al. 2022). There have been far fewer studies, however, examining the microbiota in fish skin (Gomez and Primm 2021, Wang et al. 2023, Berggren et al. 2022). Understanding the composition of fish skin microbiomes and the dynamics in response to environmental conditions will help establish their associations with health and disease (Berggren et al. 2022, Debnath et al. 2023) and in turn, optimize aquaculture practices (Palladino et al. 2021, Sánchez-Cueto et al. 2023). To date, there has been relatively little exploration of how skin microbiomes are affected by water physicochemical or biological stressors.

We identified 103 studies on marine and freshwater fish skin microbiomes with publicly available data (as of December 2022). These studies were carried out in a range of aquaculture systems and water physicochemical conditions. The experimental approaches in DNA extraction methods, amplicon sequencing depth, data analysis, and metadata availability also differed across these studies, making microbiome comparisons difficult. Furthermore, there were differences in data analysis pipelines may result in different ASV/OTU count tables, which can influence rates of false positives or false negatives (Olson et al. 2020) and the sensitivity and specificity of ASV/OTU detection (Prodan et al. 2020). Different indices to assess beta diversity to measure community similarities take into consideration different properties of the community composition that are not comparable in cross-study comparisons. For example, a Jaccard index considers only the presence/absence of taxa; a Bray–Curtis dissimilarity matrix treats all members as independent units while a Weighted Uni-Frac dissimilarity matrix includes the taxonomic relationship between members (Lozupone and Knight 2005). Thus, the chosen dissimilarity matrix influences beta-diversity values and in turn conclusions (Lozupone et al. 2007, 2010, Wong et al. 2016, Fukuyama 2019).

In this study, we analysed the microbial community composition of fish skin microbiomes using 16S RNA V4 region sequence data publicly available in the National Center for Biotechnology Information (NCBI) database. We retrieved 36 studies where the sequencing data and associated metadata were sufficiently robust for the proposed analyses. We evaluated similarities and differences in fish skin bacterial microbiota that correlate with fish taxonomy, water physicochemical variables, and rearing conditions. We show that closely related fish species host microbiomes dominated by similar bacterial taxa, supporting previous results (Brooks et al. 2016). We also identify differences in bacterial taxa and community structure associated with marine and freshwater fish and show the features of fish skin microbiomes that converge in different hosts when held under similar housing conditions or water physicochemical conditions. We furthermore emphasize the importance of standardizing, or at least adopting a more consistent approach in sequencing analysis to better enable comparisons in the characterization of microbial communities of the fish skin across studies and environmental conditions.

## Methods

### Literature search strategy and paper data selection

We applied a systematic approach to select studies for conducting our analysis to investigate the effects of species and environmental parameters on fish skin microbiome bacterial community structure. Our search strategy consisted of mining the literature for data sets using the following keywords: 16S V4 MiSeq (“fish skin” and “microbiome” | “microbiota”) published before 2023 and in English from Google Scholar. Our inclusion criteria were: (a) studies had publicly available 16S rRNA amplicon sequencing data, (b) the sequence data included the V4 hypervariable region, and (c) studies were for bony fish skin microbiomes (i.e. excluding rays, sharks and lungfish). We used only studies with sequenced V4 region of the 16S rRNA to allow for nucleotide comparisons and the construction of phylogenetic trees. We filtered for studies that used the Illumina MiSeq technology—the most popular method (to maximize the number of studies included) as comparisons across different sequencing chemistries can result in different sequencing biases (Loman et al. 2012, Allali et al. 2017, Bailén et al. 2020). Preprint publications were eligible for inclusion. All manuscripts were manually screened to determine whether they met the inclusion criteria and contained active links to fish skin microbiome datasets.

### Data quality assessments and data processing

Datasets were first filtered and validated to be derived from fish skin microbiomes or negative sequencing controls as assessed by the associated metadata. Studies were discarded if they had either low-quality reads that resulted in the loss of the majority of the dataset (overall read Phred score < 10; < 90% chance base pairs are correctly sequenced); insufficient metadata to determine the origin of sequences (as occurred for some studies where it was not possible to determine whether the samples were fish skin or water samples), or the study used reads that after quality trimming did not overlap resulting in only partial coverage of the 16S V4 region. For each of the studies included in this analysis, standard sequencing adaptors and primers available as part of the bbmap bioinformatics toolkit (v38.91) were removed and reads quality trimmed with a minimum overall Phred score of 10 using BBDuk (Bushnell 2013). Following quality trimming, the DADA2 (v1.26.0) package in R was used to error correct, dereplicate, pseudo pool amplicon sequence variants (ASVs) (which allows for the retention of rare ASVs at low abundances across samples otherwise discarded as spurious due to its low abundance within a single sample) for combined sample inference. Reads were then merged into ASVs, with ASVs shorter than 248 base pairs (V4 region ∼ 254 base pairs) and chimaeras discarded (Callahan et al. 2016). Using a novel method, all ASVs were aligned using BLAST (v2.13.0) (Altschul et al. 1990) against a dataset containing only the V4 region (McMurtrie et al. 2022). ASVs from all studies could then be trimmed to the V4 region only and merged, otherwise not possible due to differences in variable regions sequences (e.g. V3–V4 and V4–V5 become just V4, allowing comparisons). This in turn allowed for phylogenetic-based comparisons between studies. Trimmed ASVs were then assigned to taxa using DADA2 against the SILVA database silva_nr99_v138.1 (Quast et al. 2013). ASVs identified from eukaryotes, mitochondria, chloroplasts, or not present in at least two samples from the same study were discarded. This removed potentially anomalous taxa present in a study only once. All studies were subsequently merged into one phyloseq object for ease of downstream analysis (McMurdie and Holmes 2013). ASVs identified in the negative sequencing controls were removed from all studies using the Decontam R package (v1.18.0) prevalence method at a 0.5 threshold (Davis et al. 2018). This removed potentially contaminating ASVs from the combined phyloseq object ensuring there was no bias, as not all studies published negative sequencing controls. Datasets were further filtered to removed samples with low ASV abundance and low numbers of reads. This was done as these simple microbiomes when compared with highly complex microbiomes may obscure trends in microbial community structure making cross-comparisons difficult. This was done by retaining samples where the total number of reads was between the 2.5 and 97.5 percentile (keeping the middle 95%). Additionally, ASVs summarized by abundances falling within the bottom fifth percentile for each fish species across the entire dataset were deemed rare taxa and removed (i.e. the top 95th percentile of most abundant ASVs per fish species were retained only). The best phylogenetic tree substitution model for the 16S phylogenetic tree ASVs from all the merged studies was determined using IQ-TREE (v2.2.0.3) using the Bayesian information criterion (Kalyaanamoorthy et al. 2017). Maximum-likelihood trees were constructed using IQ-TREE to determine the most likelihood phylogenetic tree (Nguyen et al. 2015) and added to the phyloseq object for downstream analysis. Scripts to reproduce this process can be found here: https://github.com/ash-bell/fish_16S_metastudy_public.

### Amplicon analysis

The effects of different physicochemical factors on ASV abundance were analysed using R (v4.2.2) (R Core Team 2021). Host lineages were determined by querying TaxIDs from the NCBI taxonomic database (Schoch et al. 2020) and stored as a dendrogram using the dendextend package (v1.17.1) (Galili 2015). ASV abundance was normalized as relative abundance within a sample. Phylogenetic distance matrices were determined and ordination plots were constructed using phyloseq (v1.42.0) (McMurdie and Holmes 2013), vegan (2.6–4) (Oksanen et al. 2022) and UniFrac (vegan) (Lozupone and Knight 2005) packages. All plots were made using either ggplot2 (v3.4.0) (Hadley Wickham 2016), pheatmap (v1.0.12) (Raivo Kolde 2019), ggpubr (v0.5.0) (Alboukadel Kassambara 2022), and/or ggh4x (v0.2.3) (Teun van den Brand 2022) packages and the tidyverse (v1.3.2) (Wickham et al. 2019) package. Statistical test applied to calculate the difference between dissimilarity matrixes (PERMANOVA) were performed using the adonis2 function in the vegan package. A pairwise PERMANOVA test was performed using the pairwise.adonis2 function from the pairwise.adonis package (Pedro Martinez Arbizu 2018) (v0.4). All reported tests were performed with PERMANOVA or pairwise PERMANOVA had an adjusted *P*-value of < .001. Cultivation groups with only one replicate were not included in pairwise comparisons as the one replicate study became the sole weighting in a group, leading to potential bias. Scripts for this process can be found here: https://github.com/ash-bell/fish_16S_metastudy_public.

## Results

### Papers chosen for analysis

**Figure 1.**
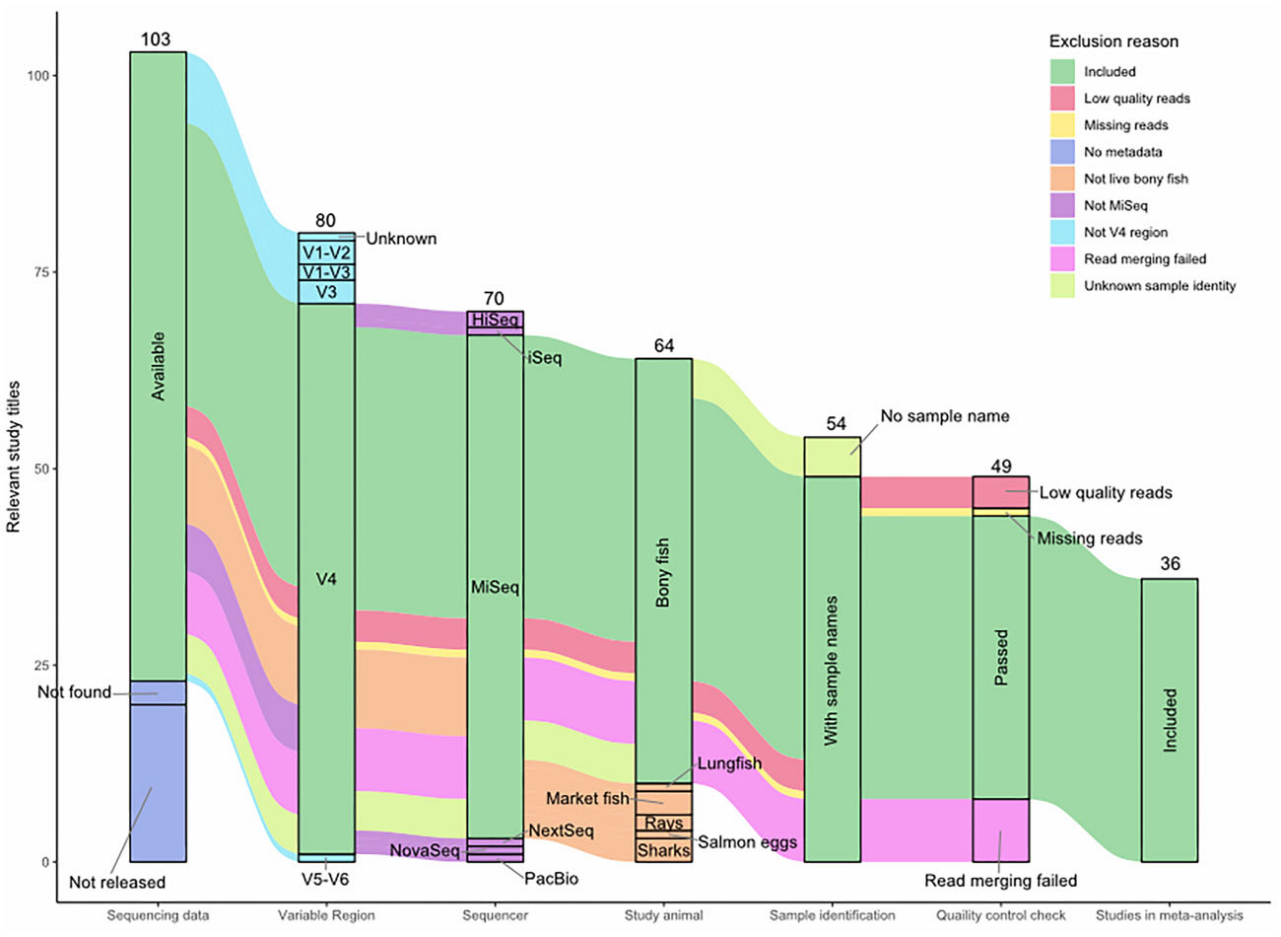
Distribution of studies analysed in the current study. 103 published studies containing relevant and original data on fish skin microbiomes were retrieved and filtered down to a final 35 unique BioProjects from 36 published manuscripts. From left to right, the number of studies that were removed for each quality assessment criteria. Some studies passed initial quality control steps that assessed for availability of 16S rRNA V4 fish skin microbiome data but were subsequently discarded downstream for the numbers and reasons shown.

Our database search for fish skin microbiome studies resulted in 290 manuscripts. The full documents were screened for applicability to this study, filtering for 16S V4 region fish skin microbiome data that were publicly available, as described above (see the sections “Methods” and “Literature search strategy and paper data selection”). This resulted in 103 manuscripts with original data on fish skin microbiomes (Fig. 1). Only studies on bony fish were included with studies on elasmobranchs including sharks (three), rays (two), and lungfish (one) excluded due to their distant phylogeny. One teleost study was discarded as it was on eggs only. Another 23 studies were discarded because there was no sequencing data released (this included four studies providing accession numbers but with no associated data). A further 10 studies were discarded as the sequencing was not based on the V4 region, including one study that did not indicate which region was used. Six studies were also excluded as they did not use MiSeq sequencing technologies (one having a discrepancy between the sequencing instrument used in the methods versus their metadata). Three further studies were excluded as they examined dead fish obtained from markets or food processing plants, thus were likely contaminated with microbes from harvesting, handing and/or processing. A further five studies had to be removed because the associated metadata did not allow for their identify as fish samples to be confirmed. This resulted in 49 studies that were suitable for inclusion within our meta-analysis (Fig. 1).

We then assessed these 49 studies for the sequencing read quality, excluding any studies that did not pass our basic quality control steps (Fig. 1). This resulted in the removal of eight of these studies because sequencing reads did not merge (due either to low base pair quality resulting in the truncation of reads, or discrepancies between overlapping regions preventing finding consensus sequences); three studies because there were so few reads per sample that error rates could not be estimated, therefore preventing error correction; and one further study because the number of forward and reverse reads differed, suggesting an incomplete dataset (Fig. 1). Studies were also removed from the analysis if no ASV was present more than twice in samples from the same study, indicating little to no diversity. Two published studies used the same BioProject number and were thus treated as a single study. This reduced the final total to 36 studies for our meta-analysis (Table 1).

**Table 1.** List of fish skin microbiome studies with publicly available sequence data used within this manuscript. Laboratory (tank)-based aquaculture systems include flow-through (with unspecified water treatment), treated flow-through (with microbial sterilization occurring before water input) or untreated flow-through (with no prior microbial sterilization and the outflow is discarded). A recirculating aquaculture system (RAS) is where the water is reused after passing through a (bio) filter system. Environmental (wild) refers to fish caught in natural freshwater and marine water bodies (e.g. streams, rivers, and seas). Outdoor aquaculture refers to ponds or sea cages exposed to the natural environment.

No.	NCBI BioProject	Species studied	Number of samples	Reference	Water condition	Cultivation type(s)	Recorded physicochemical factors
1	PRJDB10074	Rainbow trout (*Oncorhynchus mykiss*)	3	Takeuchi et al. (2021)	Freshwater	Tank; RAS	Temperature
2	PRJEB22688	Atlantic salmon (*Salmo salar*)	74	Uren Webster et al. (2018)	Freshwater	Wild	
3	PRJEB29173	Arctic char and cod (*Salvelinus alpinus, Gadus* sp.)	127	Hamilton et al. (2019)	Freshwater	Wild	Conductivity
4	PRJEB30953	Atlantic salmon (*Salmo salar*)	95	Uren Webster et al. (2020)	Freshwater	Wild, tank; treated flowthrough	Temperature
5	PRJEB38845	Rainbow trout (*Oncorhynchus mykiss*)	12	Terova et al. (2021)	Freshwater	Tank; flowthrough	Temperature, pH, dO_2_
6	PRJEB46984	Tilapia (*Coptodon rendalli, Tilapia sparmanii, Oreochromis shiranus*)	32	McMurtrie et al. (2022)	Freshwater	Outdoor aquaculture pond	
7	PRJNA323592	Tambaqui (*Colossoma macropomum*)	40	Sylvain et al. (2016)	Freshwater	Tank	Temperature, pH, dO_2_, conductivity
8	PRJNA416707	Atlantic salmon (*Salmo salar*)	7	Minniti et al. (2019)	Saltwater	Untreated flowthrough	Temperature, salinity
9	PRJNA419057	Various coral reef fish	135	Chiarello et al. (2018)	Saltwater	Wild	
10	PRJNA448853	Clownfish (*Amphiprion clarkii)*	61	Pratte, Patin et al. (2018)	Saltwater	Tank; RAS	Temperature, salinity, pH
11	PRJNA453531	Common snook (*Centropomus undecimalis*)	31	Tarnecki et al. (2019)	Saltwater	Wild, tank; RAS	Temperature, pH, dO_2_, salinity
12	PRJNA531247	Various Mediterranean teleosts	102	Scheifler et al. (2019)	Saltwater	Wild	
13	PRJNA560003	Various River Jordan fish	74	Krotman et al. (2020)	Freshwater	Wild	Temperature, pH, dO_2_, conductivity
14	PRJNA562087	Nile tilapia (*Oreochromis niloticus*)	6	Akter et al. (2021)	Freshwater	Outdoor aquaculture pond	
15	PRJNA575053	Seabass (*Dicentrarchus labrax*)	70	Rosado et al. (2019)	Estuarine	RAS	
16	PRJNA579553	Red snapper (*Lutjanus campechanus*)	31	Tarnecki et al. (2022)	Estuarine	Tank; treated flowthrough	Temperature, pH, dO_2_, salinity
17	PRJNA586895	Nile tilapia (*Oreochromis niloticus*)	2	Akter et al. (2021)	Freshwater	Outdoor aquaculture pond	
18	PRJNA596590	Prussian carp (*Carassius gibelio*)	33	Kashinskaya et al. (2021)	Freshwater	Wild	
19	PRJNA597066	Spotted robust triplefin (*Forsterygion capito*)	39	Montenegro et al. (2020)	Saltwater	Wild	
20	PRJNA599608	Stegastes damselfishes (*Stegastes leucostictus, Stegastes adustus*)	56	Xavier et al. (2020)	Saltwater	Wild	
21	PRJNA656561	Orbicular batfish (*Platax orbicularis*)	18	Le Luyer et al. (2021)	Saltwater	Tank; RAS	Temperature
22	PRJNA663352	Atlantic salmon (*Salmo salar*)	70	Bledsoe et al. (2022)	Saltwater	Tank; RAS	Salinity
23	PRJNA664785	Common snook (*Centropomus undecimalis*)	37	Tarnecki et al. (2021)	Saltwater	Tank; RAS	Temperature, pH, dO_2_, salinity
24	PRJNA667752	Rohu (*Labeo rohita*)	9	Kawser et al. (2022)	Freshwater	Outdoor aquaculture pond	
25	PRJNA687505	Seabream and seabass (*Sparus aurata, Dicentrarchus labrax*)	247	Rosado et al. (2021)	Estuarine	Outdoor aquaculture pond	
26	PRJNA692072	Seabream (*Sparus aurata*)	4	Palladino et al. (2021)	Saltwater	Wild; seacages	
27	PRJNA714685	Eurasian carp (*Cyprinus carpio*)	104	Berggren (2021)	Freshwater	Wild	
28	PRJNA741392	European seabass (*Dicentrarchus labrax*)	84	Rosado et al. (2022)	Estuarine	Outdoor aquaculture pond	
29	PRJNA748412	Various sparids	86	Scheifler et al. (2022)	Saltwater	Wild	
30	PRJNA756005	Caribbean cleaner goby (*Elacatinus evelynae*)	44	Pereira et al. (2023)	Saltwater	Wild	
31	PRJNA759847	Zebrafish (*Danio rerio*)	6	Wakeman et al. (2021)	Freshwater	Tank; treated flowthrough	Temperature
32	PRJNA763808	Discus fish (*Symphysodon aequifasciata*)	3	Huang et al. (2022)	Freshwater	tank	Temperature, pH, dO_2_
33	PRJNA792590	Striped catfish (*Pangasianodon hypophthalmus*)	38	Chen et al. (2022)	Freshwater	Tank	Temperature
34	PRJNA826829	Atlantic salmon (*Salmo salar*)	87	Lorgen-Ritchie et al. (2022 )	Freshwater, Saltwater	Tank; RAS, Wild; seacages	Temperature, conductivity, pH
35	PRJNA838496	Guppy (*Poecilia reticulata*)	55	Kramp et al. (2022)	Freshwater	Tank; RAS	Temperature

Some studies used in the meta-analysis had over 100 000 reads per sample but on average we found 20% of reads did not pass quality control filters, with some studies discarding over 50% of reads due to their low quality. Other studies comprised of samples with around 20 000 reads of which few were discarded during quality control steps. Using Good’s coverage of 100% (Good 1953), (indicating that no ASV appears in any samples only once) suggests fish skin microbiomes on average (median) reached saturation at 75 unique ASVs (212 unique ASVs, 95th percentile) and (median) 7245 reads (36 460 reads; 95th percentile) indicating that the majority of bacterial species fish skin microbiome are likely captured with these numbers.

In total, our phyloseq object contained 8003 bacterial taxa in 1922 samples across 36 different studies, comprising 98 different fish species and sampled from a range of culturing systems and environmental conditions. We investigated the bacterial composition present across these fish species and assessed for interrelationships between their skin microbiomes and a range of environmental water conditions/parameters.

### Bony fish skin have similar bacterial taxa that diverge at a bacterial order level

**Figure 2.**
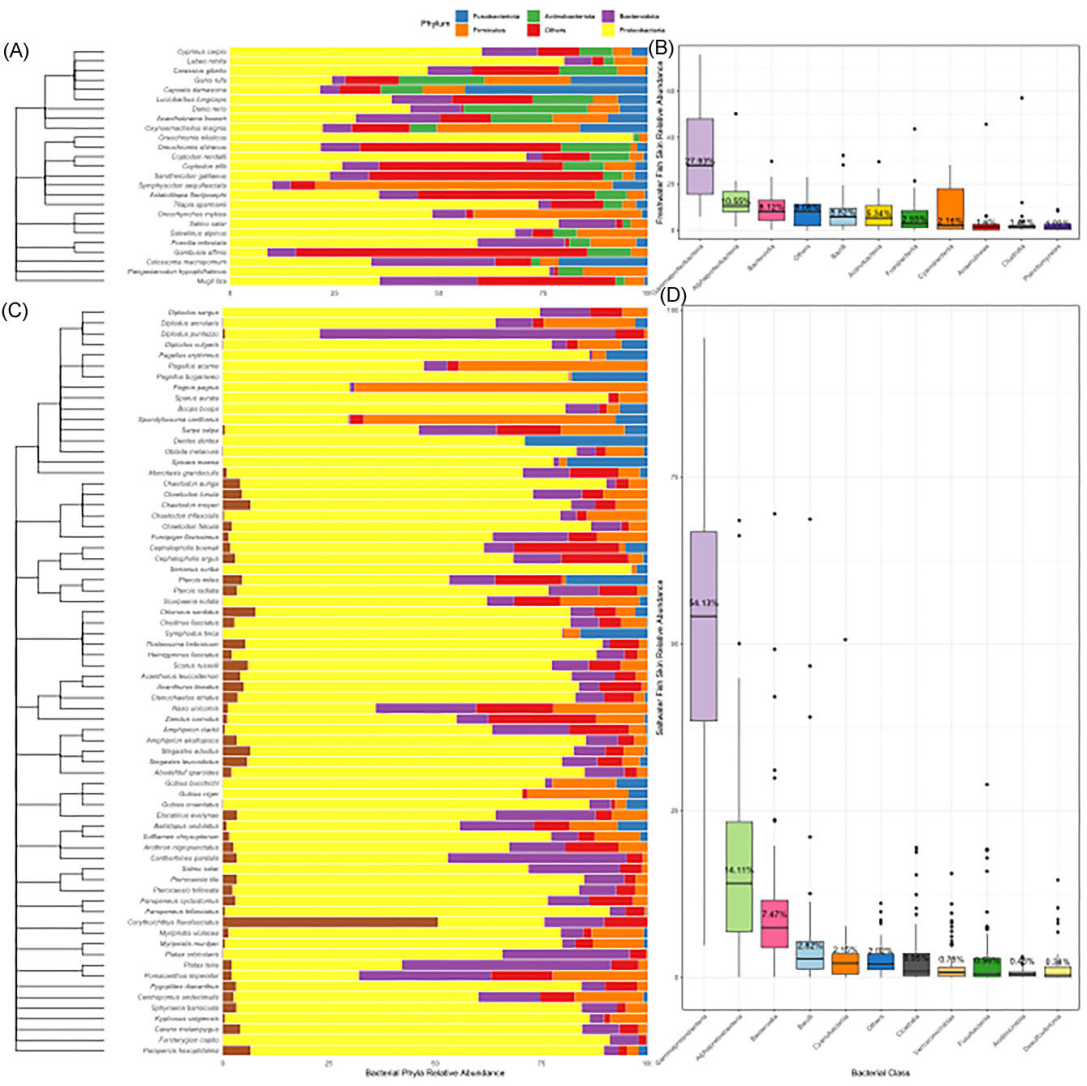
Relative abundance of fish skin microbiome bacterial community compositions at an ASV level, grouped by: (A) freshwater fish bacterial phylum level; (B) freshwater fish bacterial class level; (C) saltwater fish bacterial phylum level; and (D) saltwater fish bacterial class level.

Analysis of 98 different fish species indicated that the bacterial phylum Proteobacteria and class Gammaproteobacteria were the most abundant bacterial taxa in fish skin microbiomes. These occurred in all fresh and saltwater fish skin microbiomes analysed (Figures S1 and S2, Supporting Information). Fish skin microbiomes were clearly different in fresh versus saltwater environments, and we therefore analysed freshwater fish separately to marine species throughout this manuscript. The bacterial phylum Firmicutes was also highly prevalent in fresh (found in 24/25 species) and saltwater fish (found in 55/57 species) (Fig. 2; Figure S3, Supporting Information). When comparing the similarities of saltwater fish to other salt water fish and freshwater fish to other freshwater fish, we observed that saltwater fish exhibit greater similarities with each other than freshwater fish do with one another. All saltwater fish skin microbiomes contained the bacterial order Enterobacterales and family Vibrionaceae, with 68 out of 71 fish species analysed containing the genus *Vibrio* (Figure S1, Supporting Information). Freshwater fish skin microbiomes appeared more diverse at both a bacterial order and family taxonomic level compared with saltwater fish (Figure S1, Supporting Information). In freshwater fish, Burkholderiales and Enterobacterales were the most abundant bacteria at an order level, and *Aeromonas* the most prevalent genus (Figure S1, Supporting Information). Fish skin microbiome community compositions at a genus level were weakly correlated with host lineage (freshwater fish 5% correlation; saltwater fish 6% correlation; Figure S4, Supporting Information) and showed variations between studies at a fish order level (Figures S5, S6, and S7, Supporting Information).

### Fish cultured in different environments have different fish skin microbiome communities

**Figure 3.**
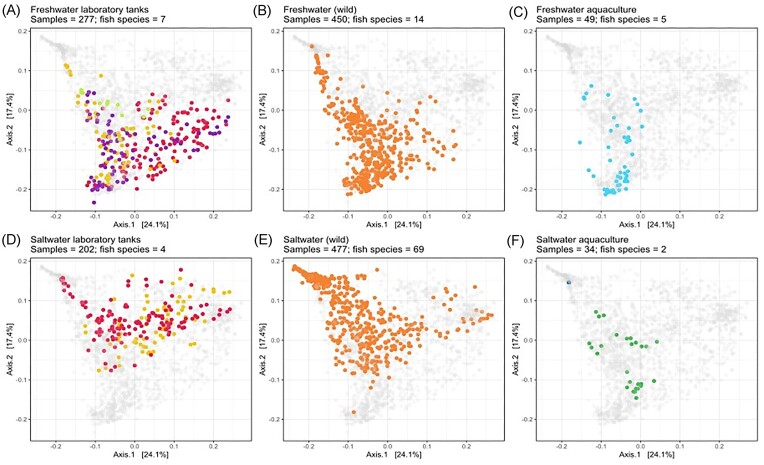
PCoA of a Weighted UniFrac dissimilarity matrix at the ASV level grouped and colour coded by cultivation system in which the fish were sampled or maintained.

Fish skin microbiome community compositions for fish cultivated in different environments had clear differences in their composition (*R*^2^ = 0.12, fresh and saltwater, *P* < .001) (Fig. 3, Tables 2 and 3). Fish skin microbiomes were most similar between fish sampled in the wild and those in outdoor aquaculture systems (sea cages or ponds) (*R*^2^ = 0.01 freshwater, 0.03 saltwater, *P* < .001). The greatest differences were seen between freshwater fish held in laboratory flow-through systems versus all other freshwater systems (Tables 2 and 3). Skin microbiomes of fish held in recirculating aquaculture system (RAS) tanks were most similar to fish from wild conditions (*R*^2^ = 0.06 within freshwater, 0.04 within saltwater, *P* < .001), followed by other tank systems with unspecified water sources (*R*^2^ = 0.09 within freshwater, *P* < .001) and treated flow-through systems (*R*^2^ = 0.20 within freshwater, *P* < .001). Outdoor aquaculture systems were most similar to fish from wild conditions, followed by tank (*R*^2^ = 0.07 within freshwater, *P* < .001), and RAS system (*R*^2^ = 0.14 within freshwater, 0.10 within saltwater, *P* < .001), and the most different to flow-through systems (*R*^2^ = 0.17 within freshwater, *P* < .001).

**Table 2.** PERMANOVA and pairwise PERMANOVA *post hoc* test results comparing different freshwater fish culturing conditions from Fig. 3. *R*^2^ indicates how well the variables fit the PERMANOVA model. Cultivation systems with only one study were removed in pairwise comparisons due to potentially biased study weighting. All statistical tests have a *P* (adjusted) value of < .001.

Freshwater system 1	Samples	Studies	Freshwater system 2	Samples	Studies	*R* ^2^
Overall PERMANOVA	707	18	NA			0.12
Outdoor aquaculture pond	49	4	Wild	381	6	0.01
Tank	81	3	Wild	381	6	0.02
Tank; treated flowthrough	62	2	Wild	381	6	0.06
Wild	381	6	Tank; RAS	122	3	0.06
Outdoor aquaculture pond	49	4	Tank	81	3	0.07
Tank	81	3	Tank; RAS	122	3	0.09
Outdoor aquaculture pond	49	4	Tank; RAS	122	3	0.14
Tank	81	3	Tank; treated flowthrough	62	2	0.15
Outdoor aquaculture pond	49	4	Tank; treated flowthrough	62	2	0.17
Tank; treated flowthrough	62	2	Tank; RAS	122	3	0.20

**Table 3.** PERMANOVA and pairwise PERMANOVA *post hoc* test results comparing different saltwater fish culturing conditions from Fig. 3. *R*^2^ indicates how well the variables fit the PERMANOVA model. Cultivation systems with only one study were removed in pairwise comparisons due to potentially biased study weighting. All statistical tests have a *P* (adjusted) value of < .001.

Saltwater system 1	Samples	Studies	Saltwater system 2	Samples	Studies	*R* ^2^
Overall PERMANOVA	713	14	NA			0.12
Wild	477	7	Wild; sea cages	27	2	0.03
Tank; RAS	132	4	Wild	477	7	0.04
Tank; RAS	132	4	Wild; sea cages	27	2	0.10

### Physicochemical factors influencing fish skin microbiome community structure and diversity

**Figure 4.**
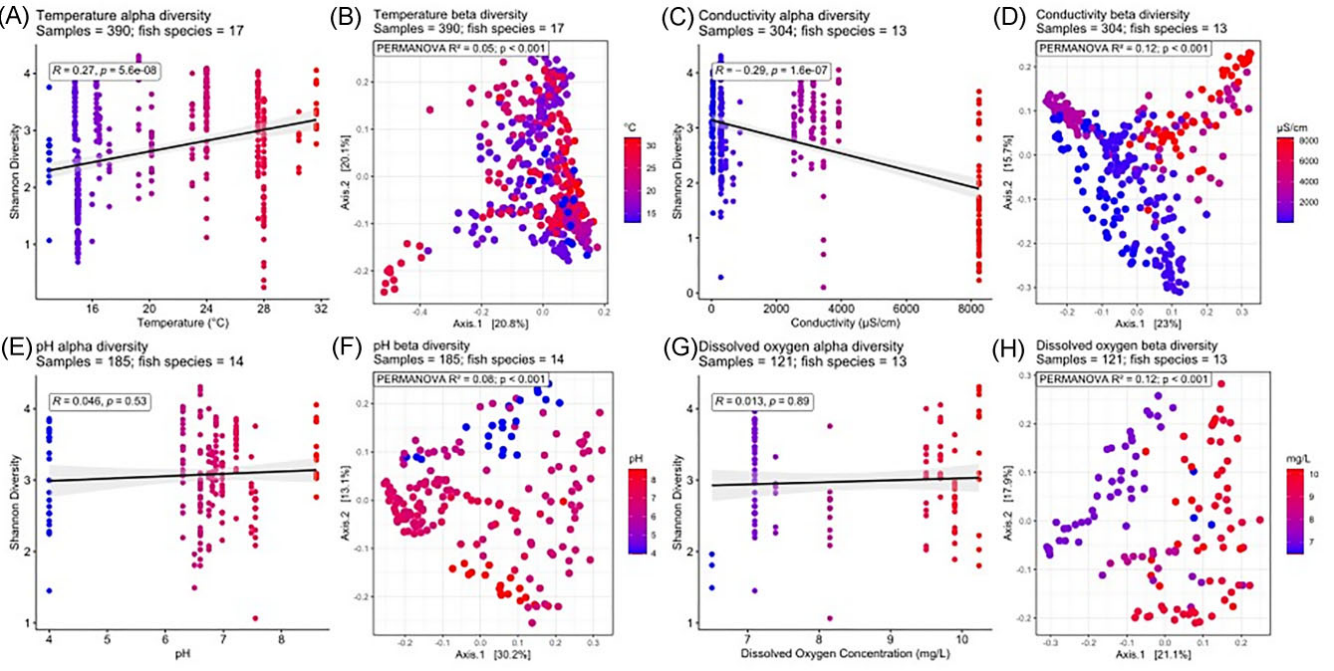
Alpha and beta diversity of freshwater fish skin microbiomes associated with features of water physicochemistry. Shannon (alpha) diversity is correlated using a linear regression model against associated physicochemical factors and correlated using Spearman’s correlation. Beta diversity is coloured according to associated physiochemical data using a principal coordinate analysis (PCoA) from a Weighted UniFrac dissimilarity matrix at an ASV level. (A) and (B) temperature, (C) and (D) conductivity, (E) and (F) pH, and (G) and (H) dO_2_ concentration.

**Figure 5.**
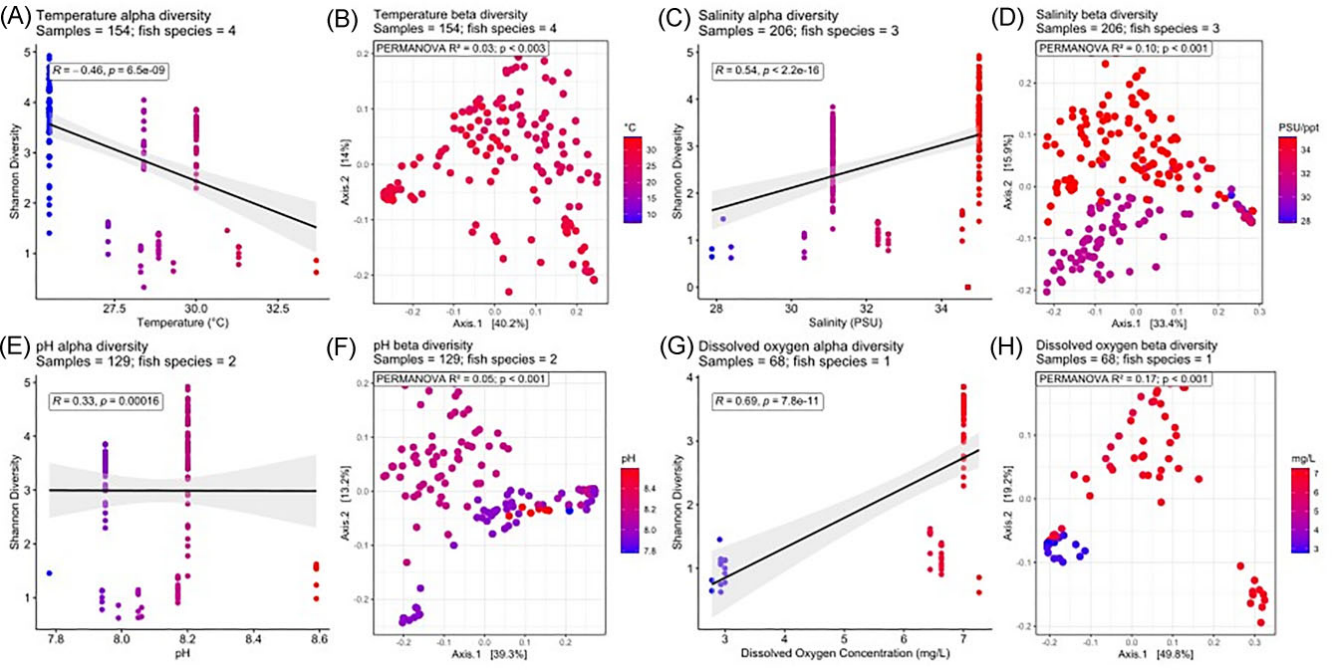
Alpha and beta diversity of saltwater fish skin microbiomes associated with recorded physicochemical factors. Shannon (alpha) diversity is correlated using linear regression to associated physicochemical factors and correlated using Spearman’s correlation. Beta diversity is coloured according to associated physiochemical data using a principal coordinate analysis (PCoA) from a Weighted UniFrac dissimilarity matrix at an ASV level. (A) and (B) temperature. (C) and (D) conductivity, (E) and (F) pH, and (G) and (H) dO_2_ concentration.

Some water physicochemistry parameters, specifically temperature, salinity, pH, and dissolved oxygen (dO_2_), correlated with fish skin microbiome bacterial community compositions. Alpha diversity in freshwater fish skin microbiomes was positively correlated with temperature (*R* = 0.27, *P* < .001) and negatively correlated with conductivity (*R* = −0.29, *P* < .001) (Fig. 4). Alpha diversity in saltwater fish skin microbiomes showed a negative correlation with temperature (*R* = −0.46, *P* < .001), but a positive relationship with salinity (*R* = 0.54, *P* < .001) and pH (*R* = 0.33, *P* < .001), with no apparent associations with dO_2_ concentration between 2.5 and 7.5 mg/l (Fig. 5).

Both fresh and saltwater fish skin microbiome beta diversity correlated with temperature, salinity, pH, and dO_2_ suggesting fish skin microbial community compositions are influenced by these factors (Fig. 4). dO_2_ concentration was the most strongly correlated physicochemical factor (*R*^2^ = 0.12 within freshwater, 0.17 within saltwater, *P* < .001) followed by salinity (*R*^2^ = 0.12 within freshwater, 0.10 within saltwater, *P* < .001), pH (*R*^2^ = 0.06 within freshwater, 0.05 within saltwater, *P* < .001) and then temperature (*R*^2^ = 0.05 within freshwater, 0.03 within saltwater, *P* < .001) (Figs 4 and 5). However, as only a few of the studies provided accompanying physicochemical metadata, this meant that only a low diversity of fish species was included in the water physicochemical comparisons. In addition, we emphasize that many studies only sampled one fish in the same conditions limiting confidence in these assessments on the effect of these physicochemical factors (Figure S8 and Table S1, Supporting Information).

### Variation in the skin microbial composition of Atlantic salmon (*Salmo salar*), a case study

As host factors likely confound attempts to qualify and quantify the influence of water physicochemistry on fish skin microbiomes, we investigated the variation in the skin microbiome for studies conducted on the Atlantic salmon (*Salmo salar*), the fish species for which there was the highest number of independent studies (*n* = 5, Table 1); one study, however, that had to be discarded due a low number of ASVs. Atlantic salmon skin microbial composition differed significantly (*R*^2^ = 0.34, *P* < .001) between studies, even after accounting for differences in salinity (the only water physicochemical factor recorded for all studies). This indicates factors other than host species and salinity (such as fish strain, differences in sampling procedure or DNA extraction) play a role also in determining fish skin microbiome community composition for this species (Figure S9, Supporting Information).

We also investigated whether temperature affected the skin microbiome of Atlantic salmon grown in freshwater (data not recorded for saltwater studies) albeit there were only two studies for this analysis (Figure S9, Supporting Information). Here, we found the abundance of 109 ASVs comprising 69 genera of bacteria significantly correlated negatively or positively with temperature changes (vegan; env.fit; *P* < .001 cutoffs) (Figures S10 and S11, Supporting Information).

## Discussion

Fish skin microbiomes play a crucial role in maintaining fish health, however, the bacterial composition of these microbiomes and their interplay in response to environmental parameters are not well-understood. Here, we use a meta-analysis framework to analyse data from publicly available studies to investigate the commonalities and differences in bacterial taxa in fish skin microbiomes across a range of freshwater and marine fish species from both cultivated and wild conditions. We investigated how host phylogeny and the environment influence these microbial assemblages and identify bacterial taxa that may constitute core elements of the fish skin microbiome. We also make suggestions and recommendations for future studies relating to sequencing and data curation to better enable future comparisons of microbiomes between studies.

### Bacterial composition of fish skin microbiomes

All fish skin microbiomes were dominated by the Proteobacteria phylum and Gammaproteobacteria class, as has been identified previously in the literature (Boutin et al. 2014, Lokesh and Kiron 2016, Krotman et al. 2020). Divergence of fish skin microbiome community compositions occurred at the level of bacterial order, with no bacterial genus present in all fish species. Saltwater fish skin microbiomes shared greater similarities, compared to that observed within freshwater fish. Supporting this observation, microbiomes of marine damselfish (*Stegastes leucostictus*) separated by 225 km were seen to have similar skin beta-diversities (Xavier et al. 2020) and only 3% of the microbiome beta-diversity in coral reef fish was explained by reef locations (Chiarello et al. 2018). In contrast the beta diversity of the skin microbiome of freshwater European catfish (*Silurus glanis*) from river sites separated by 200 km differed significantly (Chiarello et al. 2019). These findings may reflect more consistent physiochemical properties across marine environments than in freshwaters. Supporting this, a 1-year time series study on *Scomber japonicus* skin microbiomes showed little variation in marine water physicochemistry (Minich et al. 2020a), whereas a study of Amazonian teleost species from freshwater sites were separated by clear hydrochemical gradients (Sylvain et al. 2019). These findings support that fish skin microbiomes community compositions are influenced by the physiochemistry of their environment in both marine and freshwater environments.

Common genera found in the skin microbiomes of freshwater fish included *Acinetobacter, Aeromonas, Ralstonia, Sphingomonas* (all Proteobacteria), and *Flavobacterium* (Bacteroidota); and in saltwater fish included *Alteromonas, Photobacterium, Pseudoalteromonas, Psychrobacter*, and *Vibrio* (all Gammaproteobacteria class). For some of the fish orders analysed there was only one species representative in this meta-analysis (especially for Gadiformes, Istiophoriformes, and Centrarchiformes), increasing the likelihood that the presence of any given bacterial genera may be missed and in turn the microbiome bacterial assemblages more universal than suggested.

Genera such as *Aeromonas, Acinetobacter, Flavobacterium*, and *Vibrio* contain species with known pathogenicity to fish. *Aeromonas hydrophila*, e.g. is ubiquitous within freshwater environments and is associated with diseases including bacterial haemorrhagic septicaemia and epizootic ulcerative syndrome (Lategan et al. 2004, Chen et al. 2022). *Flavobacterium psychrophilum* is the primary agent of bacterial cold-water disease and rainbow trout fry syndrome, which is one of the main sources of economic loss of the salmonid industry (Duchaud et al. 2018). *Acinetobacter* spp. contain strains that are emerging as septicaemic disease-causing agents for a wide variety of freshwater fish (Malick et al. 2020), albeit they occur also in the skin microbiomes of nondiseased freshwater fish. *Vibrio* occurs commonly in the skin microbiomes of healthy saltwater fish but also include species such as *Vibrio anguillarum* that can cause septicaemia and are one of the most common saltwater fish pathogens in the aquaculture industry (Austin et al. 1995, Weber et al. 2010, Chaudhary et al. 2021). Species within the *Ralstonia* and *Sphingomonas* genera are poorly categorized especially within fish microbiomes. We cannot specify whether the genera highlighted contain these pathogens, but it is well established they can be commensals or pathobionts and can cause disease in response to host stressors (Bass et al. 2019).

The presence of bacteria such as *Sphingomonas, Psychrobacter*, and *Ralstonia* in microbiomes has received attention recently. *Sphingomonas* spp. synthesize astaxanthin, as part of the carotenoid synthesis pathway responsible for the red pigmentation in salmon and krill (Tam et al. 2021) and may act to provide some probiotic protection to fish, e.g. as shown in mortality rates in rohu (*Labeo rohita*) subjected to *V. anguillarum* infection (Chaudhary et al. 2021). *Psychrobacter* spp. includes opportunistic pathogens in both fish and humans and is the causative agent of disease in Red Sea marine fish (El-Sayed et al. 2023). However, *P. nivimaris* and *P. faecalis* have also been documented to have probiotic qualities, reducing mortality rates in juvenile turbot (*Scophthalmus maximus*) exposed to *Tenacibaculum maritimum* (see Wuertz et al. 2023). *Ralstonia* spp. include plant and opportunistic human pathogens (Waugh et al. 2010, Said et al. 2020), that occur naturally in water and soil microbiomes (Ryan et al. 2011). *Ralstonia* spp., along with *Photobacterium* and *Acinetobacter*, are dominant members of the fish gut microbiome, and may have a key role in dietary function or are transferred from fish skin during predation (Huang et al. 2020). The functional roles of these opportunists and commensals, however, are poorly understood. For bacteria such as *Alteromonas* and *Pseudoalteromonas* in the microbiome of saltwater fish, almost nothing is known about their function. The presence of common bacterial genera across widely differing species indicates some functional commonalities in the fish skin microbiome but generally this is still relatively poorly understood. This may also change in response to environmental stressors and disease. For example, the genus *Psychrobacter* contains both opportunistic pathogens and symbionts, with thus both a protective function but also a disease-causing impact (El-Sayed et al. 2023, Wuertz et al. 2023). It is well-established that when fish are stressed, their microbiomes are perturbed leading to an increase in the abundance of opportunistic pathogens, which may lead to the development of disease (Minniti et al. 2017). A greater understanding of the function of the different bacterial genera and their relationship with the host fish and how they respond to environmental stressors is clearly needed to better define their roles in health and disease.

### Relationship between fish phylogeny and skin bacterial community composition (phylosymbiosis)

Previous studies have documented a correlation between the taxonomic relatedness of fish and the similarity in their microbiomes, a concept referred to as phylosymbiosis (Brooks et al. 2016, Chiarello et al. 2018). In our analysis we found a relatively limited link between fish skin bacterial community compositions and host lineage, however, this may relate to the fact that many fish were represented by a single species. Also, within our analysis, the prevalence of bacterial genera in the skin microbiome varied for different fish orders. Illustrating this, for Salmoniformes there was largely a consistent bacterial composition and abundance reported across multiple studies, whereas in the Perciformes there was a far greater variability in the most prevalent taxa. In the Perciformes fish order, only *Photobacterium, Pseudoalteromonas*, and *Vibrio* genera were highly prevalent and abundant in all host species analysed. This disparity, however, may in part be because the most common Perciform in this analysis, *Dicentrarchus labrax*, was cultured in estuarine waters while all other Perciformes species were from saltwater environments, likely driving differences in the skin community composition for the analysis on the Perciforms. When comparing the skin microbiomes of fish from within the same host orders held within saltwater across multiple studies there was a higher degree of similarity of bacterial genera (see e.g. members from the Pomacentridae fish family; Figure S5, Supporting Information). Although some genera were common across different fish species (such as *Aeromonas* in freshwater and *Vibrio* in saltwater) some genera appeared to be limited to specific taxonomic orders, such as *Chryseobacterium* in the Salmonidae (Figure S7, Supporting Information). In studies on coral reef fish where several different fish species were analysed within the same study stronger correlations have been reported between host lineage and skin microbial community compositions (Chiarello et al. 2018, Pratte et al. 2018).

### Effect of cultivation system on fish skin bacterial community compositions

We hypothesized that the diversity and composition of fish skin bacterial communities are likely to be influenced by fish culturing systems, due to different environmental conditions. Our results indicate that overall, 12% of beta diversity was explained by fish culturing systems in fresh and also within saltwater conditions. This supports previous studies indicating that different aquaculture environments can result in different fish microbiome community structures (Minich et al. 2020b, Lorgen-Ritchie et al. 2022). Skin microbiomes in fish held in flow-through aquaculture systems differed most from all other types of fish cultivation systems (RAS, wild-caught, and outdoor aquaculture), with RAS systems more closely resembling fish captured from the wild or from aquaculture environments, which were broadly similar. These differences in skin microbiota may reflect differences in water supplied for fish in captive conditions, e.g. systems sterilized via UV and/or ozone, whereas RAS systems with biofilters only have a rich bacterial flora that will also be also in the water column.

### Water physiochemistry factors shaping fish skin microbiomes

For both fresh and saltwater conditions, difference in water physicochemical factors including salinity, temperature, pH, and dO_2_ correlated with fish skin bacterial community composition (beta diversity), with the strongest correlations seen with dO_2_ concentration, followed by salinity, pH, and with smallest effect for temperature. For freshwater fish, temperature was positively correlated with alpha diversity. No correlation was observed for pH or dO_2_, and there was a negative correlation with conductivity. For saltwater fish, alpha diversity increased with a more basic pH, higher salinity, and dO_2_ concentration but decreased at higher temperatures. Studies assessing the effect of physicochemical factors on fish skin microbiomes have similarly shown that exposure to high temperatures results in decreases in alpha diversity over time and differences in beta diversity in saltwater greater amberjack (*Seriola dumerili*) (Sánchez-Cueto et al. 2023) and in chum salmon (*Oncorhynchus keta*) (Ghosh et al. 2022). Studies on the flag cichlid (*Mesonauta festivus)* and black piranha (*Serrasalmus rhombeus)* found no effects of pH on skin microbiomes (Sylvain et al. 2019), however, in Tambaqui (*Colossoma macropomum*) (data included within this study) exposure to a low pH (pH 4.0) compared to control (pH 6.3) resulted in changes in fish skin beta diversity, but not alpha diversity (Sylvain et al. 2016). Small differences in salinity (less than 2 ppt) were not found to correlate with fish skin beta diversity in Pacific chub mackerel (*S. japonicu*s) (see Minich et al. 2020a) or for 44 different coral reef fish species (see Chiarello et al. 2018) (data included within this study). In the black molly (*Poecilia sphenops*), clear differences were seen for larger salinity shifts (13 ppt), which may relate to changes in fish osmoregulation (Schmidt et al. 2015) and in anadromous Arctic Char (*Salvelinus alpinus*) (data included within this study) alpha diversity was lower in freshwater conditions with differences in beta diversity (Hamilton et al. 2019, 2023). Studies on fish from the River Jordan (data included within this study) found differences in beta diversity in fish skin microbiomes exposed to different temperatures, dO_2_ concentrations, conductivity (salinity), and pH. Increases in alpha diversity were also observed with increased temperature, dO_2_ concentrations, conductivity (salinity), and a more basic pH. However, these physiochemical factors were substantially less correlated with alpha diversity in “corrected” skin communities (when water microbiota at the same abundance in the fish skin were removed). In contrast, in this study we only found an increasing alpha diversity relationship with increasing temperature, the most correlated physicochemical factor in Krotman et al. (2020)’s study. Our alpha diversity correlation data illustrate that salinity, temperature, pH, and dO_2_ can all influence the fish skin microbiome, but the magnitude and direction of the effect is likely dependent on the magnitude of change in the water physicochemical parameters, and/or by the physiological adaptability of the fish to the environmental conditions. Changes in water physicochemistry may also affect the water microbiome and this in turn could play a part in reshaping the skin microbiome (Krotman et al. 2020, Sylvain et al. 2020).

When considering Atlantic salmon only, different studies have reported different skin microbiomes, even when accounting for physicochemical factors such as salinity (Lorgen-Ritchie et al. 2023). Other factors, therefore, relating their maintenance or other aspects of water chemistry must play a role in determining fish skin microbiome community composition. These factors likely include salmon genetics (seed stocks), water microbiota, husbandry practice, life history and/or diet (Minniti et al. 2017, Bledsoe et al. 2022, Lorgen-Ritchie et al. 2022). Our analysis on the effect of water temperature on the skin microbiome in Atlantic salmon was extremely limited due to the available data but nevertheless the indication from that analysis was for water temperature associated changes in the abundance of 69 genera of bacteria in Atlantic salmon skin microbiomes. Further studies are thus much needed to determine what specific bacterial taxa (such as pathobionts and commensals) are enriched or depleted in fish skin microbiomes with features of water physiochemistry. This would support optimizing aquaculture systems to ensure optimal animal health, and we suggest this is especially true for closed (e.g. RAS) production systems.

### Fish skin microbiome concluding remarks

From this study, we show that the dominant taxa in fish skin bacterial microbiomes are the Proteobacteria phylum and Gammaproteobacterial class, regardless of host, environment, and water salinity. When comparing different hosts, fish skin microbiomes diverge at a bacterial order level, with some similarities between closely related species (phylosymbiosis) (Brooks et al. 2016). Fish skin microbiome differs significantly between saltwater and freshwater fish and the top genera found in salt and freshwater are completely different, with greater similarities occurring between saltwater fish. These top genera include potential pathobionts, that may cause disease when host microbiomes are perturbed, and commensals which play a crucial role in pathogenic defence and nutrient degradation likely resulting in higher host fitness. Although the type of cultivation environment influences fish skin microbiome composition, the host and other environmental factors appear more influential in determining fish skin microbiome community compositions. More studies on different fish species maintained in the same environments and across a range of physiochemical conditions are required to better determine specifics on the bacterial taxa enriched by different environmental conditions. Meta studies such as the one presented herein would notably benefit from published studies providing more comprehensive metadata relating to the fish (e.g. genetics, strains, and so on) and water physicochemistry exposure conditions. Encouraging this will expand the utility of microbiome sequencing data and allow for further investigations beyond the initial, primary, studies. Data integration of amplicon sequencing from divergent studies, reproducibility, and future comparisons would be greatly enhanced by adopting more consistent approaches in the data collection, analysis methodology, and higher standards for data management and stewardship. To help in this regard for the planning of future studies we present our recommendations for a robust data collection strategy and analysis approach in Supplementary documents to this paper (5.0 Supplementary Documents).

## Supplementary Material

fiae021_Supplemental_File
