# Supplementary material for: Influence of host phylogeny and water physicochemistry on microbial assemblages of the fish skin microbiome
Source: FEMS Microbiol Ecol. 2024 Feb 16;100(3):fiae021. doi: 10.1093/femsec/fiae021 (PMC10903987; doi:10.1093/femsec/fiae021)
Supplement: fiae021_Supplemental_File [file fiae021_supplemental_file.docx]

## Supplementary Figures, Tables and Documents


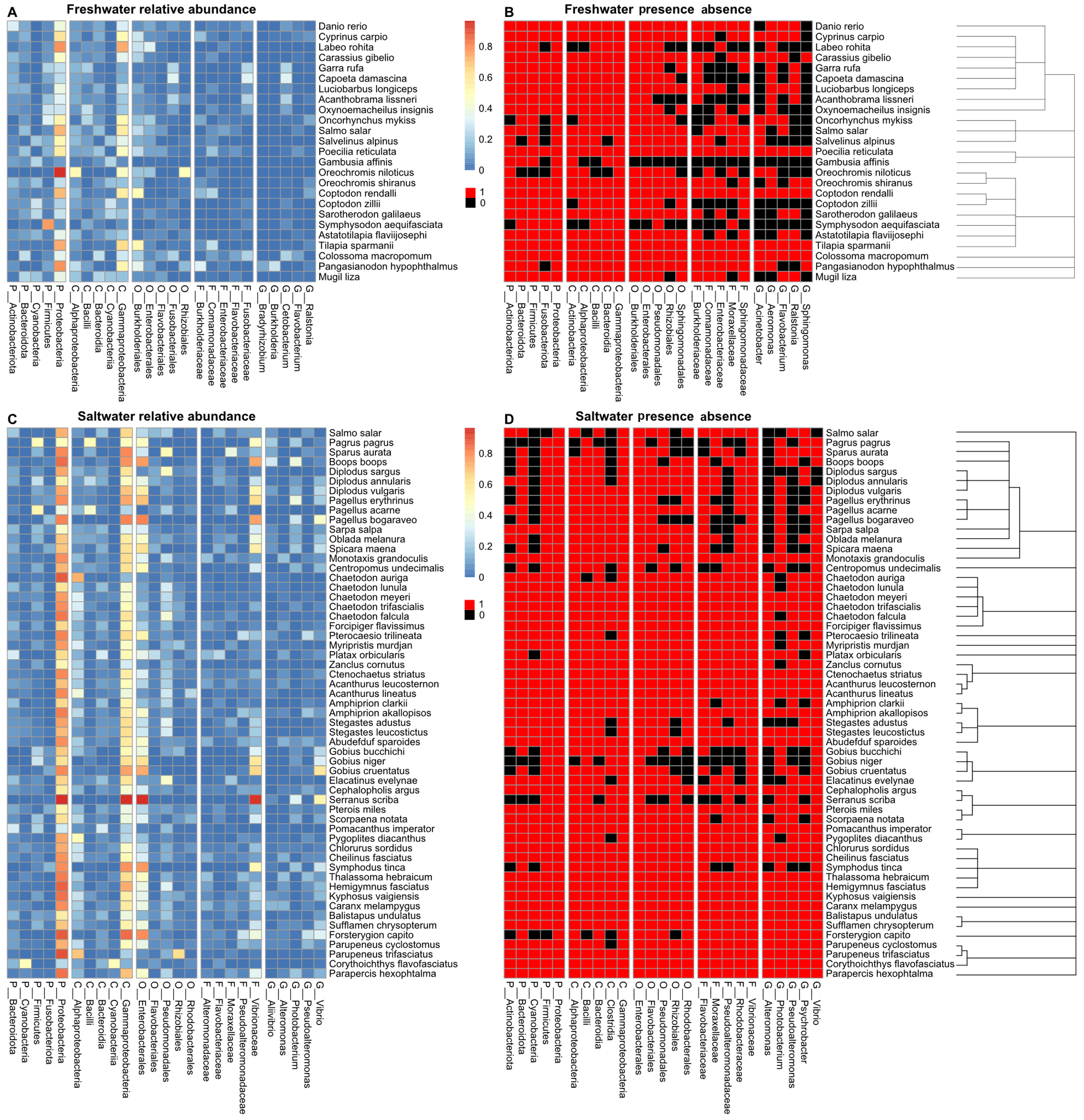


Supplementary Figure 1 ASVs abundance of the top five bacterial taxa at each bacterial taxonomic level across all bony fish species skin microbiomes ordered by fish taxonomy and categorised by: A) Freshwater relative abundance, B) Freshwater presence-absence, C) Saltwater relative abundance, D) Saltwater presence-absence.


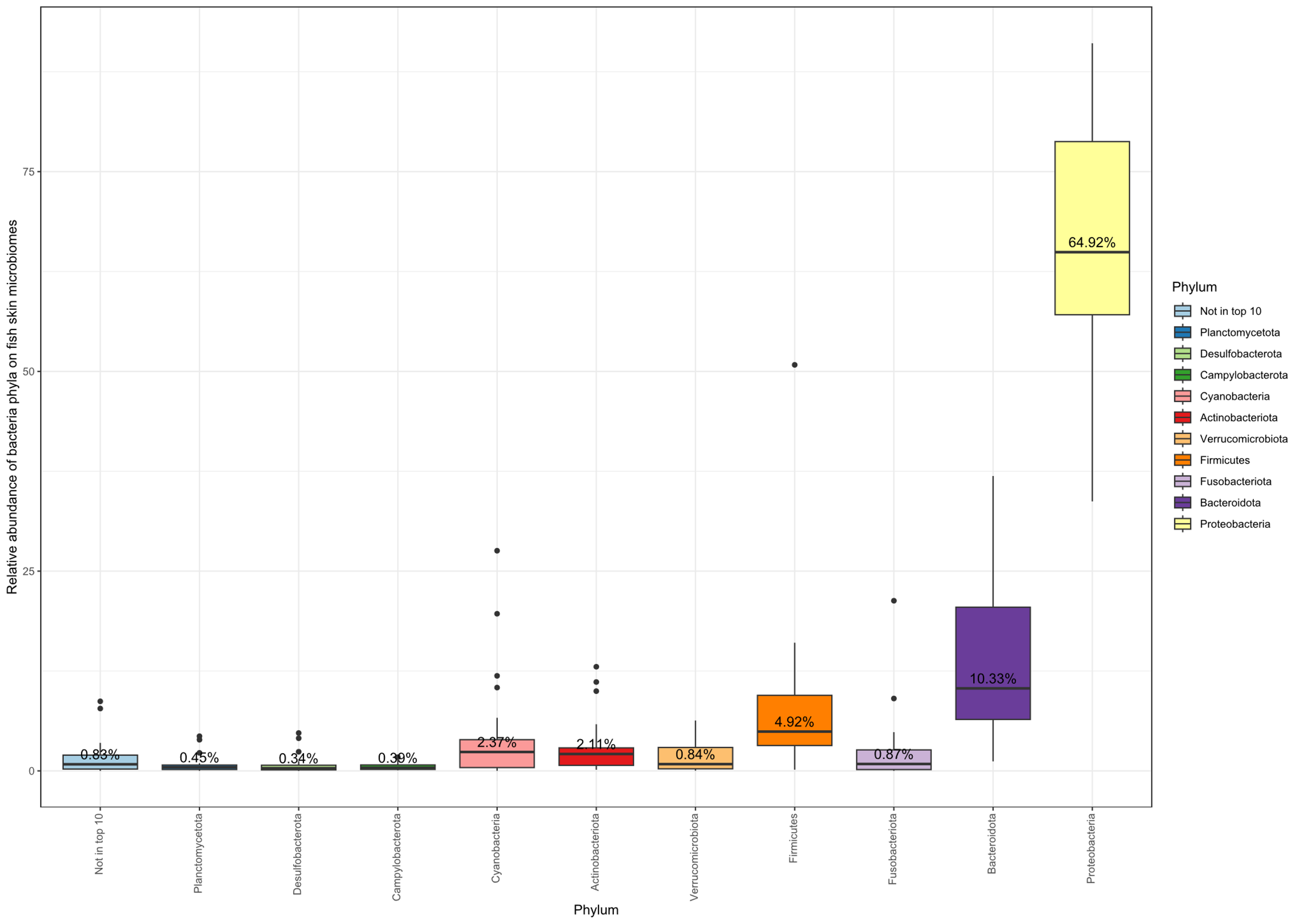


Supplementary Figure 2 Relative bacterial abundance of ASV collapsed at a phylum level of fresh and saltwater fish species’ skin microbiomes summarised by their median abundance


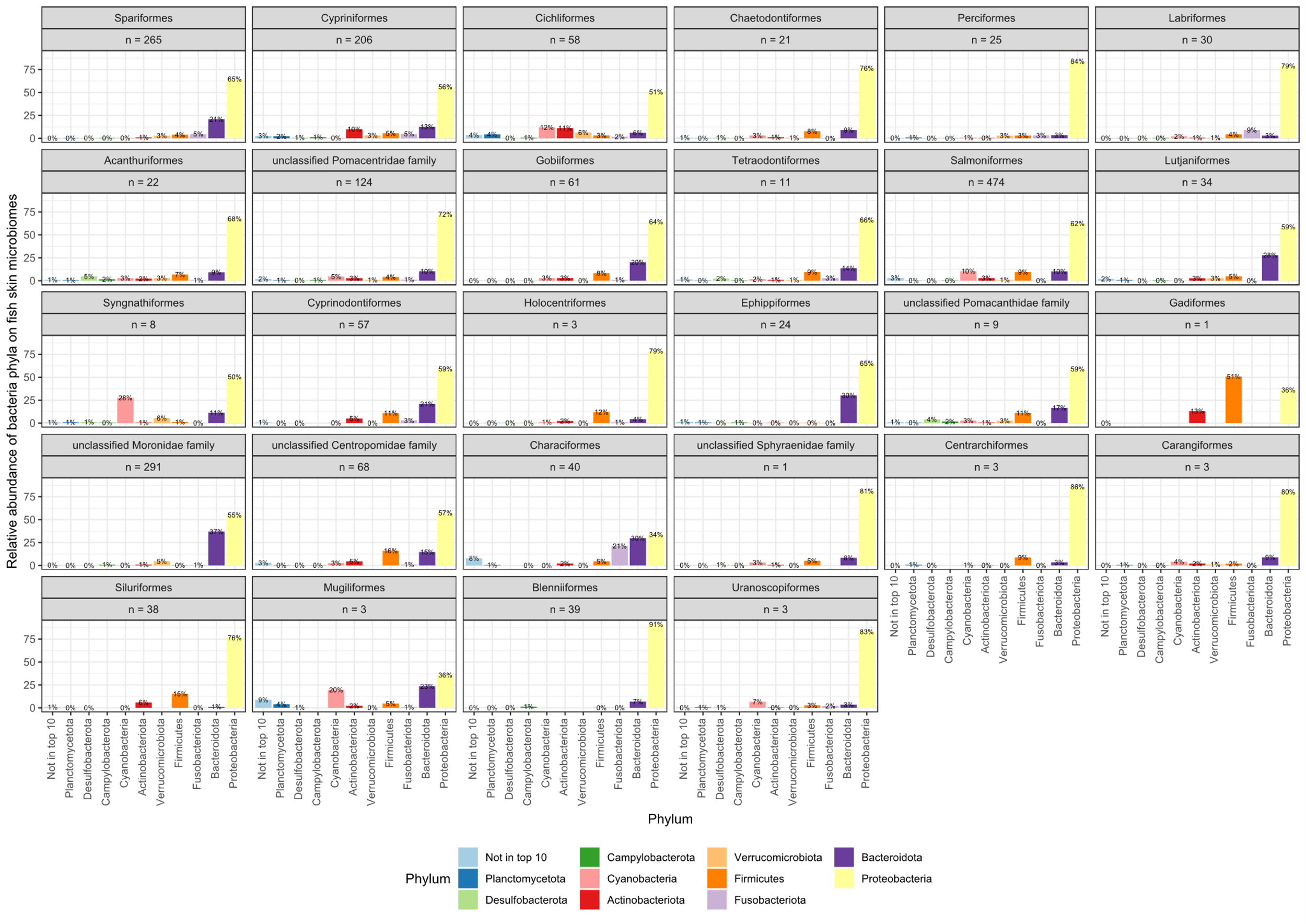


Supplementary Figure 3 Relative bacterial abundance of ASV collapsed at a phylum level grouped by various fish orders’ skin microbiomes, summarised by their median abundance.


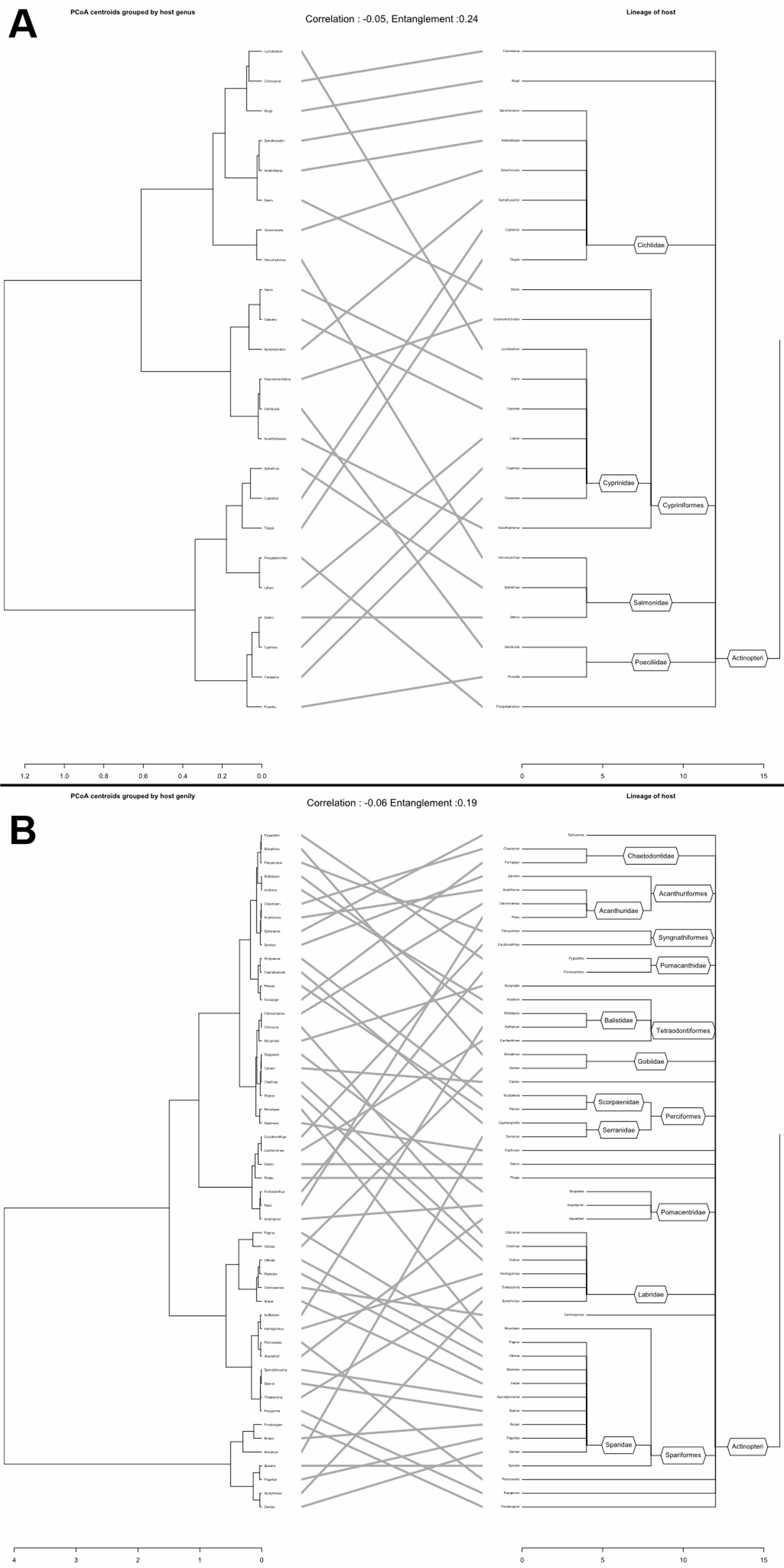


Supplementary Figure 4 Tanglegram plots of A) Freshwater B) Saltwater showing how closely related fish skin microbiomes at a genus level show a similar relatedness to host phylogeny. The bacterial community composition of fish skin microbiomes at an ASV level is determined using a weighted UniFrac dissimilarity matrix, grouped by host genus and cut using hierarchical clustering to show microbiomes that are closely related as branches on a dendrogram. Hierarchical clustered microbiomes are compared against their host lineage to determine if host microbiomes are more similar to closely related host fish. Correlation is defined as a cophenetic correlation indicating how similar two dendrogram structures are - with 1 indicating a perfect correlation. Entanglement indicates the distance dendrogram labels are to each other – with 0 indicating all dendrogram labels are directly opposite each other.


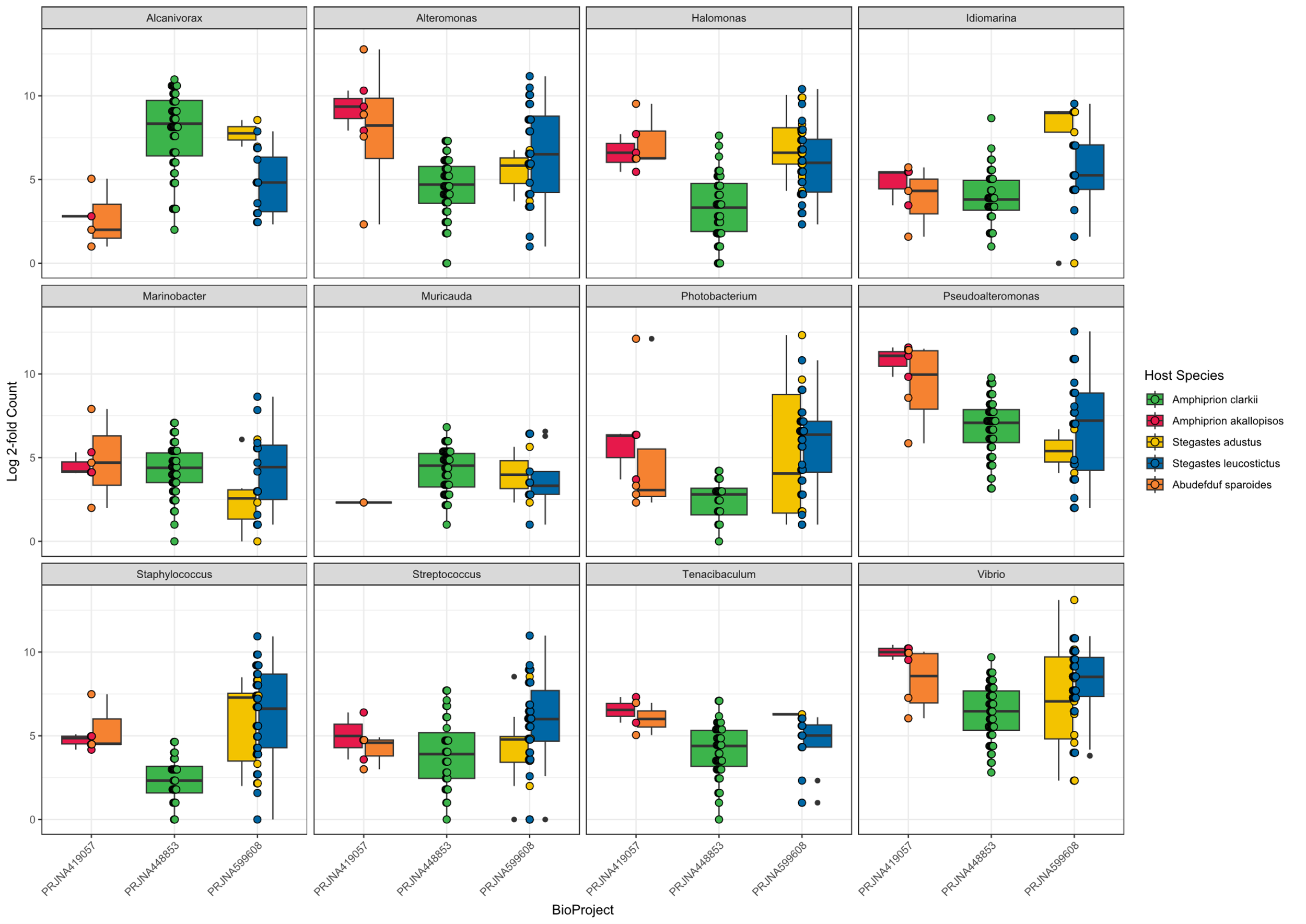


Supplementary Figure 5 Log 2-fold count of the top 12 genera of bacteria found amongst all Pomacentridae fish family within this study.


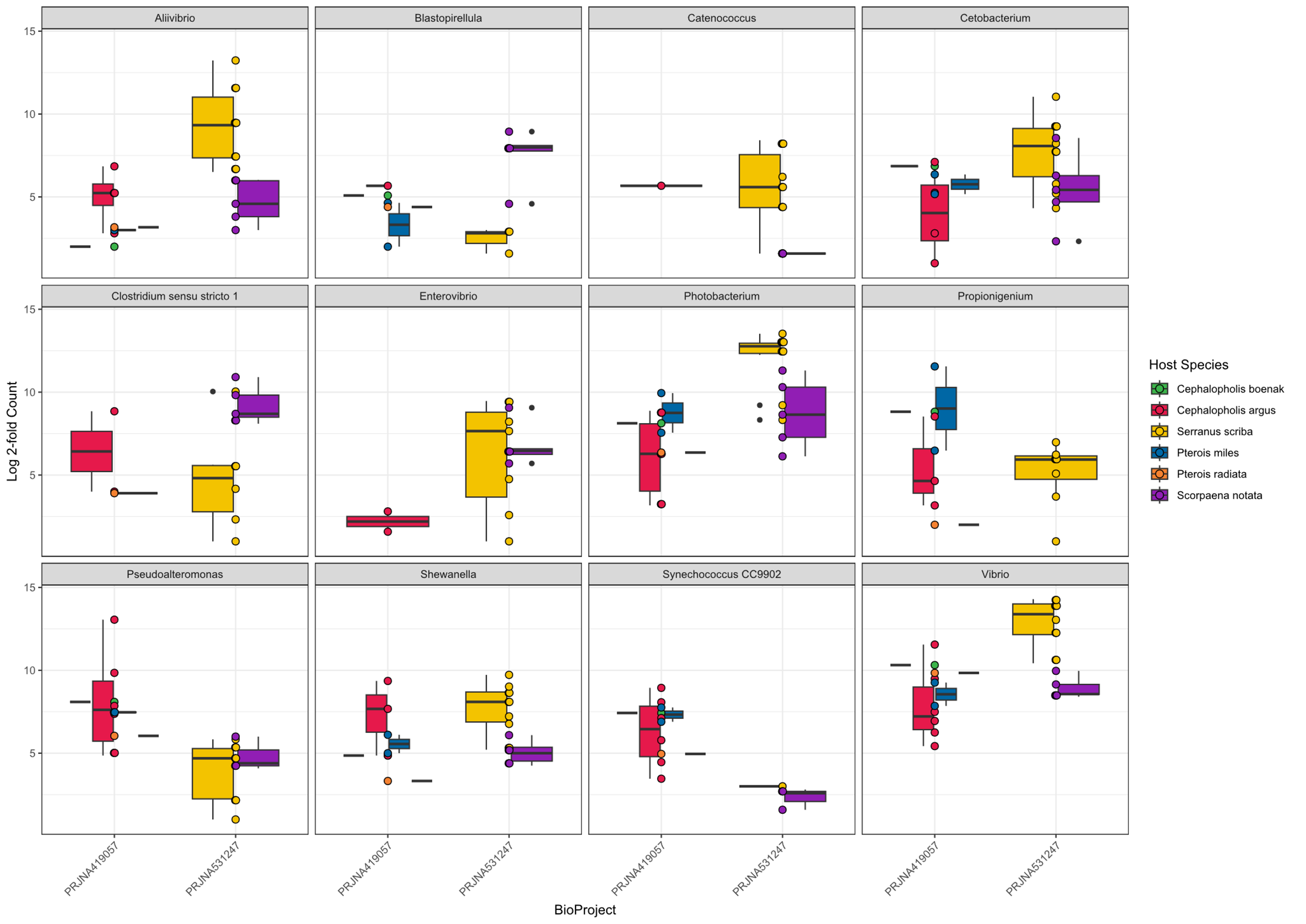


Supplementary Figure 6 Log 2-fold count of the top 12 genera of bacteria found within the Perciformes fish order within this study.


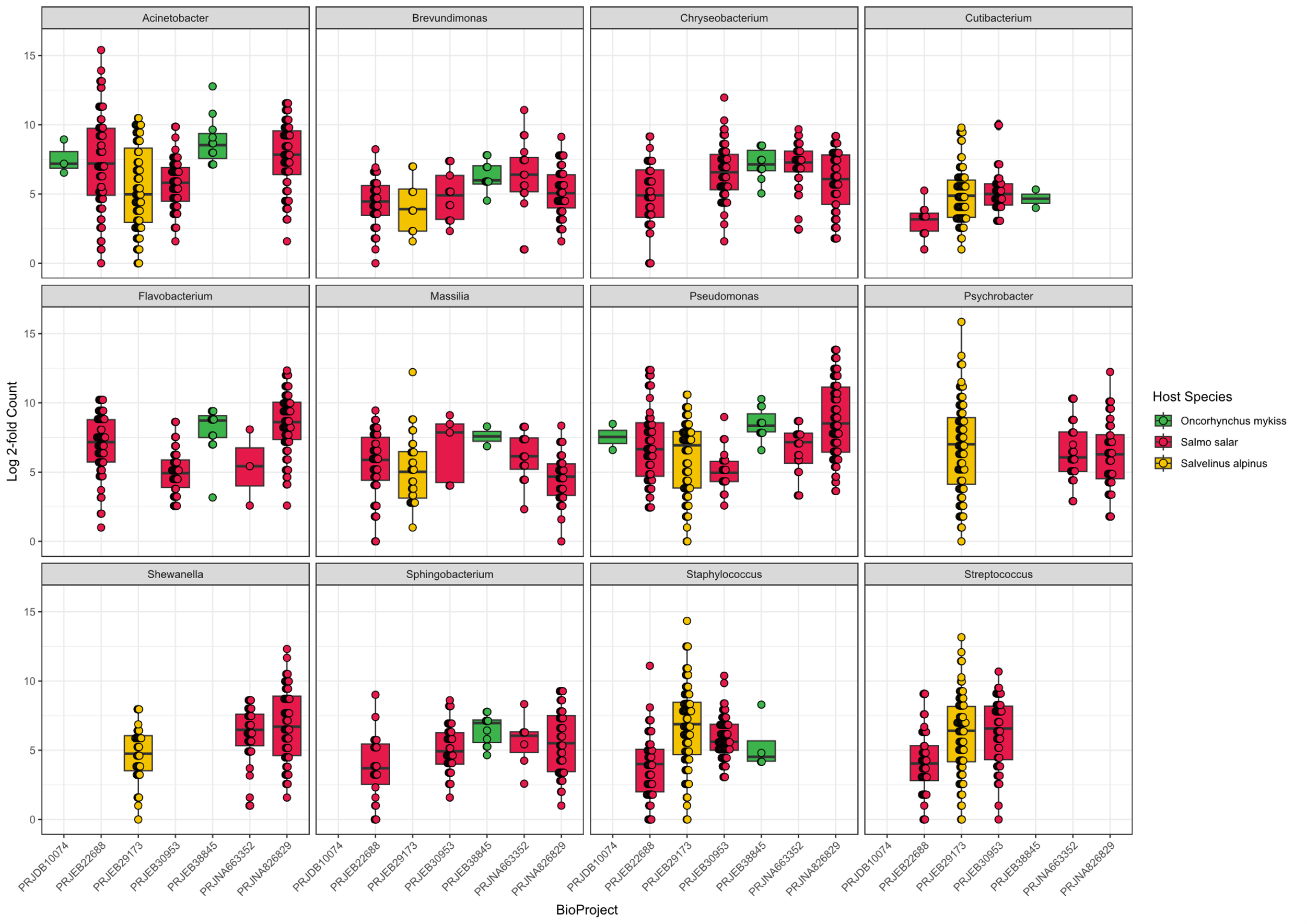


Supplementary Figure 7 Log 2-fold count of the top 12 genera of bacteria found within the Salmoniformes fish order within this study.


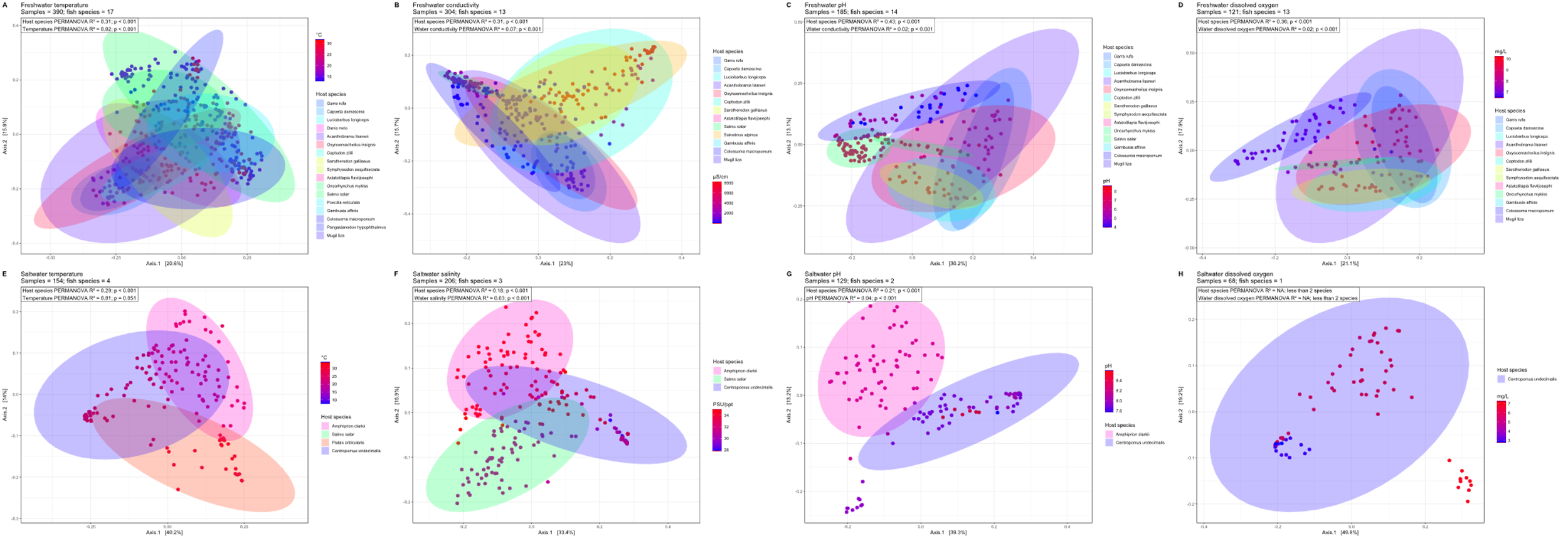


Supplementary Figure 8 PCoA of a Weighted UniFrac dissimilarity matrix at an ASV level split by environmental conditions and grouped by fish species fish. Freshwater fish skin microbiome compositions are coloured according to: A) Temperature, B) conductivity, C) pH, D) Dissolved oxygen concentration. Saltwater fish skin microbiome compositions are coloured according to: E) Temperature, F) conductivity, G) pH, H) Dissolved oxygen concentration


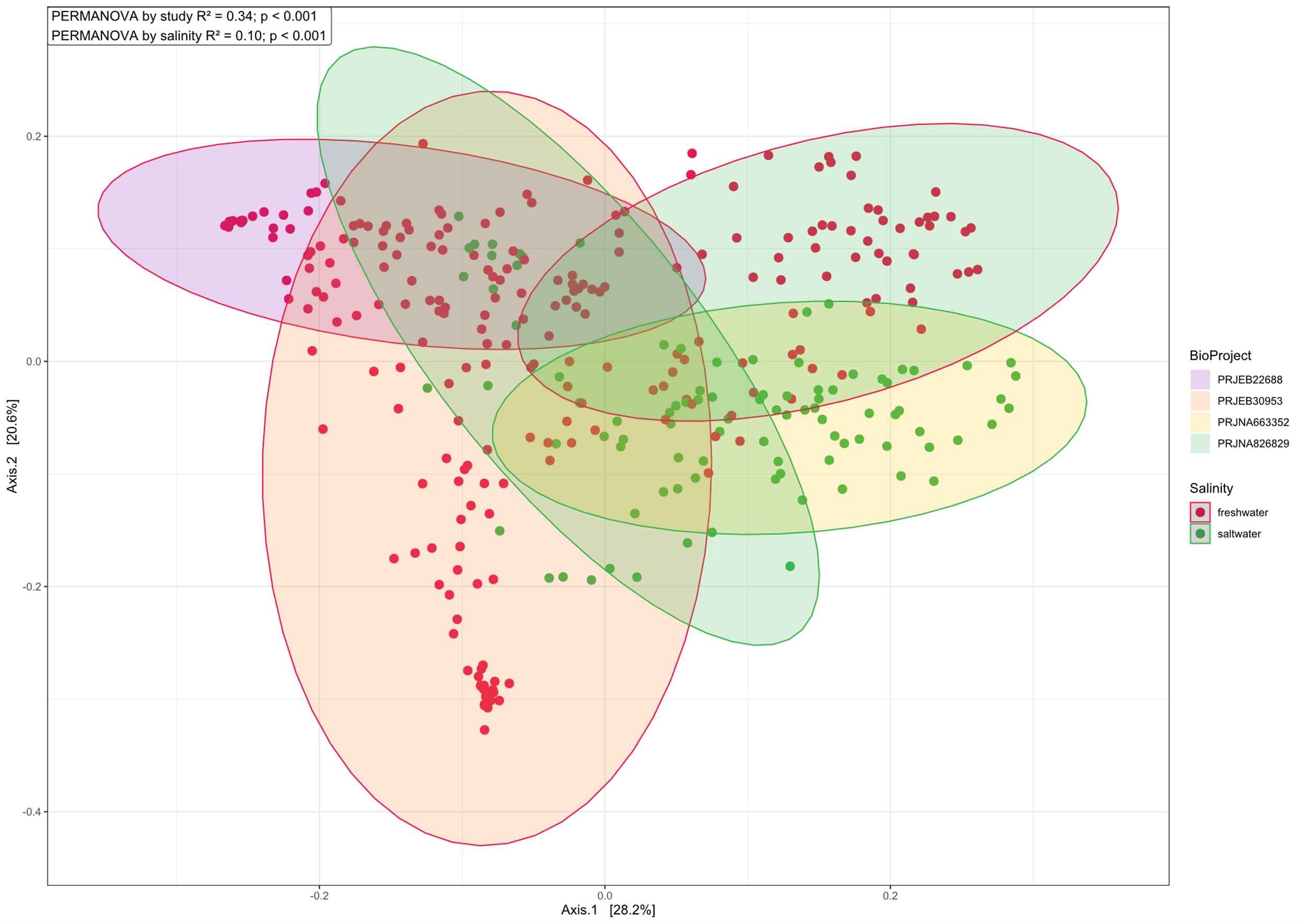


Supplementary Figure 9 PCoA of a Weighted UniFrac dissimilarity matrix at an ASV level coloured and grouped by study and salinity show significant differences of fish skin microbiomes between studies.


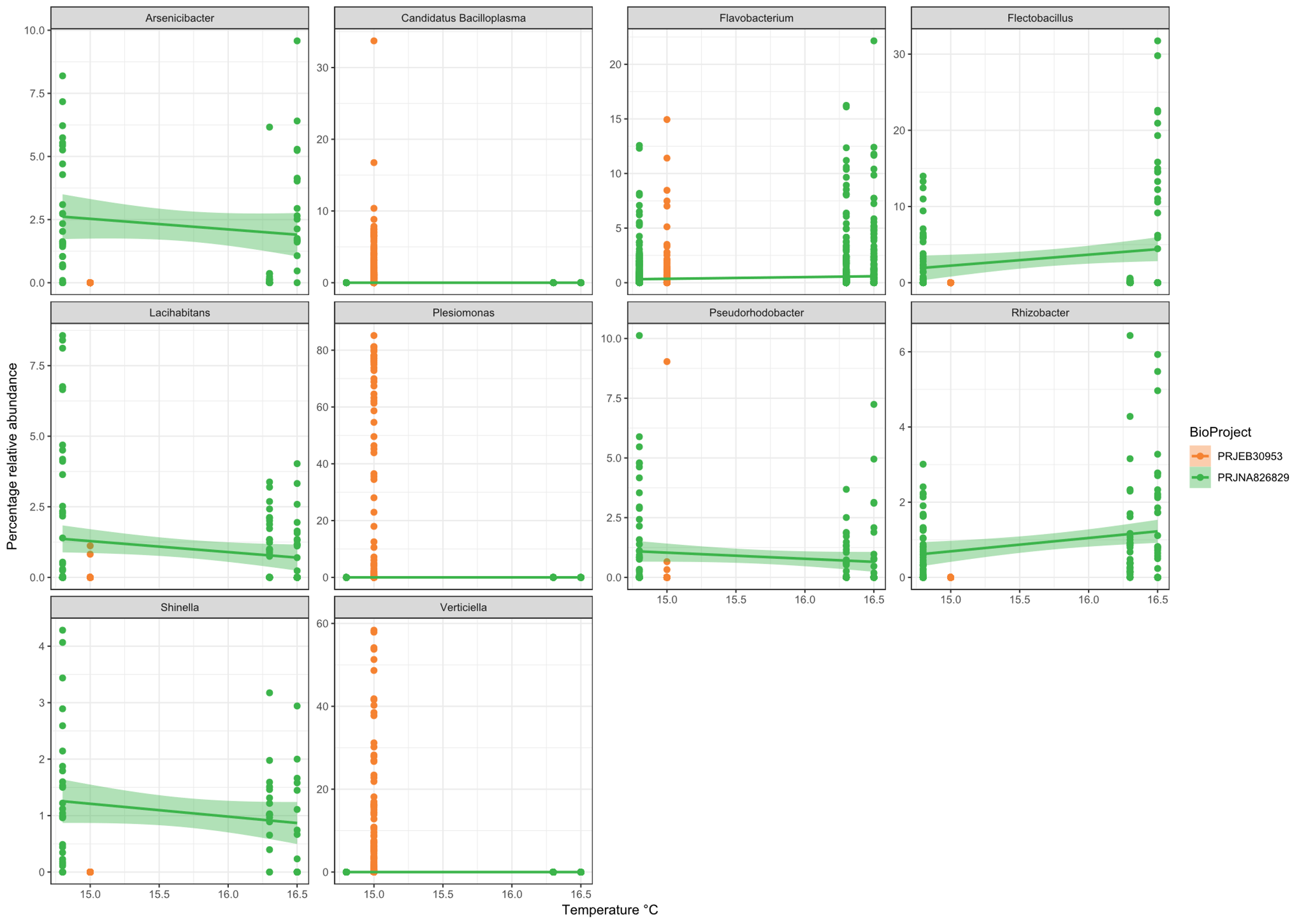


Supplementary Figure 10 Relative abundance at different temperatures of the top ten bacterial genera correlated with changing temperature in freshwater *Salmo salar.* Bacterial abundance changes associated with temperature are correlated using a linear regression model.


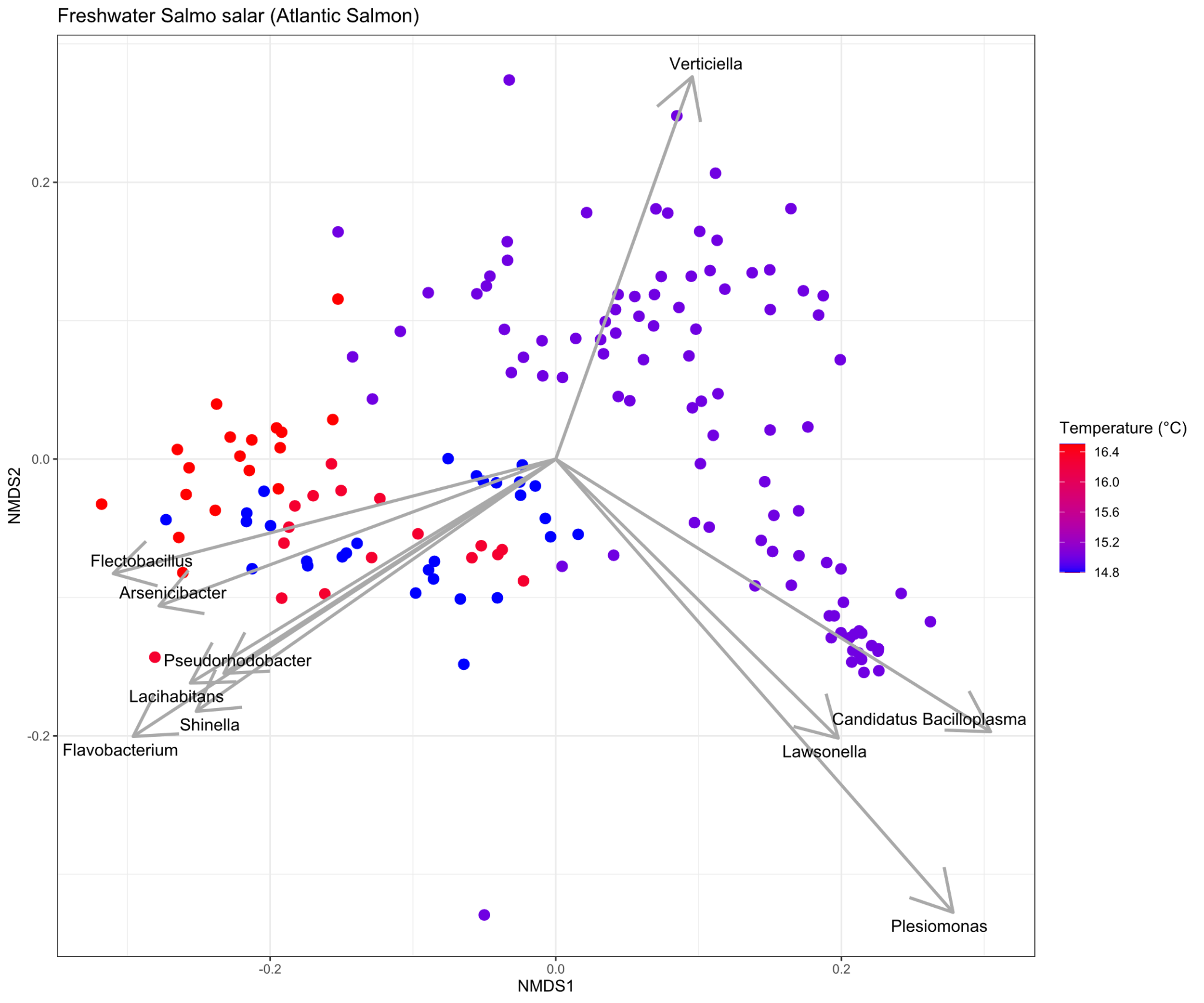


Supplementary Figure 11 NMDS of a Weighted UniFrac dissimilarity matrix at an ASV level of freshwater *Salmo salar* skin microbiomes coloured by the water temperature in which they were sampled. Arrows indicate the direction abundance of the top ten bacterial genera correlated with changing, with longer arrows indicating a stronger correlation (R^2^ value).


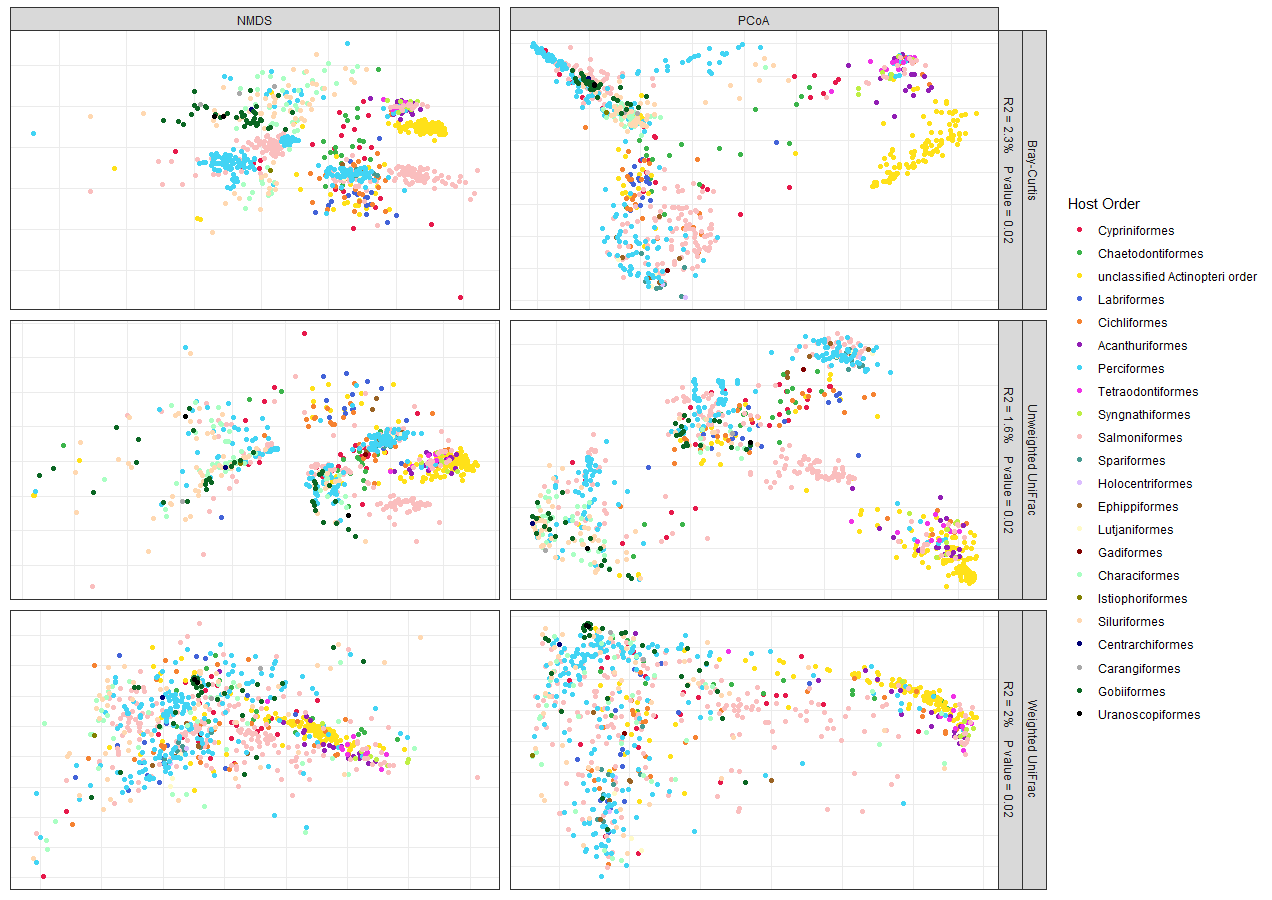


Supplementary Figure 12 Comparison of different dissimilarity matrices on host order.

Supplementary Table 1 Pairwise PERMANOVA post-hoc test results comparing different physicochemical factors separated by water condition seen in (Figure 4, Figure 5). R^2^ indicates how well the variables fit the PERMANOVA model. All statistical tests have a p (adjusted) value of < 0.001.

| Water condition | Physicochemical factor | Term | R^2^ |
| --- | --- | --- | --- |
| Freshwater | Temperature | Host species | 0.31 |
|  |  | Temperature | 0.02 |
|  | Conductivity | Host species | 0.31 |
|  |  | Conductivity | 0.07 |
|  | pH | Host species | 0.43 |
|  |  | pH | 0.02 |
|  | dO_2_ | Host species | 0.36 |
|  |  | Dissolved oxygen | 0.02 |
| Saltwater | Temperature | Host species | 0.29 |
|  |  | Temperature | 0.02 |
|  | Conductivity | Host species | 0.18 |
|  |  | Conductivity | 0.03 |
|  | pH | Host species | 0.21 |
|  |  | pH | 0.04 |
|  | dO_2_ | Host species | NA |
|  |  | Dissolved oxygen | NA |

### **5.0 Supplementary Documents**

### **5.1 Recommendations when sequencing fish skin microbiomes**

Several studies were excluded from this study due to a lack of metadata in the NCBI database (whether the samples were skin swabs, water or negative control for example). Such data in repositories are vital to promote reproducibility and transparency. Minimum metadata reporting standards have been recommended by multiple authors (Jurburg *et al.*, 2020; Stevens *et al.*, 2020; Sabot, 2022). As a minimum, data should include the type of sample (water, fish skin, negative or positive control), fish species, the culture environment (for example pond, RAS, wild-caught) and ideally also include appropriate physicochemical data - including salinity and temperature, geographical coordinates, and treatment regime (for example chemical exposure concentration or other challenge type information).

The sequencing and the types of analyses applied to that sequencing data are major factors in determining the quality and comparability of microbiome studies (Nayfach and Pollard, 2016; Wensel *et al.*, 2022). When selecting papers for this meta-analysis, we had to exclude multiple studies that resulted in the failure to merge forward and reverse reads as they no longer overlapped, largely due to low-quality towards the tail ends of reads. This is a well known issue when 300 base paired reads are used for V4 analysis (Sylvain *et al.*, 2020). We found that the 250 base pair length paired ends reads for sequencing 16S rRNA variable regions (such as the V4 region) was the most consistent and reliable. Consideration should be given to the effective sequencing length which can be reduced by the inclusion of redundant nucleotides that are typically included in amplicons to initiate sequencing on Illumina platforms. Although unmerged reads are still usable, only one direction of the reads (forward or reverse) can be used, resulting in the loss of 50% of the dataset and increasing uncertainty when assigning taxa accurately. In many cases on the Illumina MiSeq platform, the steep drop in quality at the tail of 300 base pair reads resulted in 250 base pair reads having longer lengths after quality trimming (Sylvain *et al.*, 2020). This negates the use of the 300 bp read for the capture of longer hypervariable regions, but offers a lower cost per read (20 – 25 million reads per run compared to 12 – 15), so remain applicable for shorter (< 250 base-pair) amplicons. We found that the 250 base pair length paired ends reads for sequencing 16S rRNA variable regions (such as the V4 region) was the most consistent and reliable method.

Sequencing negative controls is important as laboratory regents and DNA extraction kits can contain contaminating DNA, which critically impacts the analyse of low microbial biomass samples (Laurence, Hatzis and Brash, 2014; Salter *et al.*, 2014). Amongst the most common bacteria identified in this study, belonging to the genera *Acinetobacter*, *Flavobacterium*, *Photobacterium* and *Psychrobacter,* were found in negative controls. In our analysis, these genera still occurred after their removal using the Decontam protocol indicating they may be true members of fish skin microbiomes, or that a lack of negative controls from all studies makes detecting true contaminants difficult (only three studies had negative controls). Other studies have detected the genera *Acinetobacter*, *Ralstonia* and *Sphingomonas* as common laboratory contaminants, however the latter two were not found in our negative controls (Kulakov *et al.*, 2002; Laurence, Hatzis and Brash, 2014; Salter *et al.*, 2014). These results show contaminants could be mistaken as being common genera in fish skin microbiomes. Negative controls can be removed bioinformatically, thus their inclusion can reduce the likelihood of identifying false positive microbial members of fish skin microbiomes (Davis *et al.*, 2018).

Sequencing depth is a key factor in ensuring most taxa are captured in the sequencing process. This analysis suggests a sequencing depth minimum of 36,460 error-corrected reads should normally be deep enough to characterise fish skin microbiomes. Exceptions to this might include studies on gnotobiotic animals, for example. It is also important to consider the number of reads discarded in quality control steps, as on average we found 20% of reads did not pass quality control filters. To account for reads lost in quality control steps, we predict that approximately 44,000 (36,460 + 20%) raw paired-end reads would be sufficient in capturing total fish skin microbial diversity within the 16S V4 region.

### **5.2 Bioinformatic recommendations for analysing fish skin microbiomes**

In our meta-analysis of the published data on fish skin microbiomes, we evaluated three different dissimilarity matrixes for calculating beta-diversity, the Bray-Curtis, Unweighted and Weighted UniFrac matrices. Our findings show little difference in the ability of these methods to separate bacterial microbial community composition, however, we recommend, the Weighted UniFrac dissimilarity matrix method to determine beta-diversity (as per Lozupone and Knight, 2005; Lozupone et al., 2010; Chang, Luan and Sun, 2011; Chen et al., 2012) as microbiomes with more similar species are more likely to have similar functions (Huttenhower *et al.*, 2012; Parks *et al.*, 2015, 2017; Chaumeil *et al.*, 2020), which cannot be captured by the Bray-Curtis matrix which considers all ASVs to be universally distinct. This was particularly apparent as NMDS plots using Bray-Curtis dissimilarity matrices separated samples from different studies as completely different entities, even with the same host species in the same rearing conditions, whereas UniFrac plots clusters species from alternate studies more closely together.

Identifying taxa that are core or transient to the fish skin microbiome is challenging because of the dynamic relation of the skin across space and time and its close interrelationship with the surrounding water. The distinction between core and transient taxa however is important as core taxa are seen as essential for maintaining healthy host functions, whereas transient taxa (that may sometimes simply be a product of sampling the environment, not the host) can be argued to have a less important interaction between the host microbiomes. To address this issue Krotman et al. (2020) removed ASVs present in water samples that occur at similar abundances to those found on fish skin. This approach is similar to the methods for removing contaminated reads using Decontam R package (Davis *et al.*, 2018). This method although effective in helping to identify core microbiota may cloud understanding of the microbiome as transient microbiota nevertheless impact host skin microbiomes. Our recommendation therefore would be to retain all microbial taxa for studies on fish skin microbiomes for any comparative functional analysis.
